# Programmable multistep CRISPR gene activation via control of RNA polymerase III termination

**DOI:** 10.1126/sciadv.adt1532

**Published:** 2025-12-05

**Authors:** Anupama K. Puppala, Andrew C. Nielsen, Maureen Regan, Georgina E. Mancinelli, Renee F. De Pooter, Stephen Arnovitz, Caspian Harding, Michaele McGregor, Nikolas G. Balanis, Ryan Clarke, Bradley J. Merrill

**Affiliations:** ^1^ Syntax Bio Inc., Chicago, IL 60642, USA.; ^2^ Department of Biochemistry and Molecular Genetics, University of Illinois at Chicago, Chicago, IL 60607, USA.

## Abstract

Although genomes encode instructions for mammalian cell differentiation with rich syntactic relationships, existing methods for genetically programming cells have only modest capabilities for stepwise gene regulation. Here, we develop a sequential genetic system that transcriptionally activates endogenous genes in a preprogrammed, stepwise manner. This system uses the removal of an RNA polymerase III termination sequence to trigger both the transcriptional activation and DNA endonuclease activities of a Cas9-VPR protein, driving progression through a cascade of gene activation events. The system’s functionality in human cells, including iPSCs, enables the development of a path for cellular programming by controlling the sequential order of gene activation to influence cellular states.

## Teaser

Programming the stepwise activation of several endogenous genes is made possible in stem cells with a synthetic biology system.

## INTRODUCTION

Cellular differentiation is a sequential process in which progenitor cells progress through successive intermediate cell states toward specialized differentiated cells. The process is driven forward via multiple steps with activation and repression of a relatively small number of genes, frequently those encoding transcription factor (TF) proteins (*1*, *2*). By controlling the transcriptional activity of target genes, TFs establish cellular identity by determining the genes expressed by cells. Moreover, through the modulation of various epigenetic mechanisms (e.g., posttranslational modifications of histone, DNA methylation, and three-dimensional arrangement of the genome), TFs also influence the developmental potential of cells by restricting lineage possibilities during successive steps in a cellular trajectory.

Current approaches for genetically programming cells attempt to override the genomically encoded stepwise progression through intermediate states by forcibly expressing TFs important for a desired final cell state. Seminal discoveries demonstrated effectiveness of this approach for reprogramming somatic cells to induced pluripotent stem cells (iPSCs) (*3*), differentiating fibroblasts to myoblasts (*4*), and reprogramming nonneural cells into neurons (*5*). More recently, large-scale searches revealed TF combinations capable of programming iPSCs into a variety of other cell types, including vascular endothelial and oligodendrocyte-like cells (*6*, *7*). However, by skipping intermediate stages, such approaches may limit cellular function due to persistence of epigenetic features of the starting cell type and incomplete acquisition of target cell type features (*8*–*10*). In addition, many of the most therapeutically impactful cell types (e.g., pancreatic β cells and hematopoietic stem and progenitor cells) appear to require a stepwise process for their differentiation and have been unattainable with an all-at-once gene programming approach (*8*, *11*, *12*).

Synthetic biology solutions intending to overcome such limitations typically use molecular gates that can activate TFs and other genes in multiple, sequentially delimited steps. By combining efficient and functional individual molecular gates, synthetic gene circuits could be constructed in accordance with progression through intermediate states observed in normal cell lineages (*12*, *13*). To be effective, the core molecular gates must be step specific by remaining inactive until triggered, and gates must efficaciously activate genes once triggered. Circuits composed of these gates must efficiently trigger sequential steps in a high percentage of cells, operate within a timeframe conducive to differentiation, and have the capacity to control enough TF genes to program multiple steps.

RNA-guided nucleases and clustered regularly interspaced short palindromic repeat (CRISPR) components are attractive tools for engineering mammalian synthetic biology systems because of their inherent biochemical simplicity, specificity of DNA binding, and programmability for addressing nearly any genomic locus (*14*). Previous studies have developed methods enabling spatiotemporal regulation of spCas9 (*15*–*17*). Although regulation of the nuclease provides control over all Cas9 activities, it does not enable modular control at a per-gene level, which is mediated by the guide RNA molecule bound to Cas9 (*18*). Multiple studies have generated conditional guide RNA by manipulating the conformation of RNA with the addition of toehold base-pairing sequences or aptamers controlled by small molecules or light (*19*–*22*). Unfortunately, none of the existing systems have achieved requisite levels of specificity, efficacy, efficiency, or complexity to execute more than a two-step program in cells.

In contrast to systems using conformational changes to modify guide RNA molecules, the previously described proGuide system used Cas9-mediated editing of a DNA encoding an inactivated guide RNA as the principal molecular gate (*23*). Conceptually, separation of the triggering mechanism (DNA editing) from the activity of the molecular gate (guide RNA) provided advantages for achieving competence in the parameters essential for programming cellular differentiation. In practice, several characteristics of the proGuide system precluded it from being readily used for constructing synthetic gene circuits in human cells. To begin to meet the demands for cell programming, we systematically identified causes underlying inefficiencies in proGuides and adapted the system to drive transcriptional activity of endogenous genes. The process resulted in a highly efficient molecular gate unit, which formed the basis of synthetic gene circuits that activated multiple genes in a sequential order in individual human cells, including human iPSCs. The engineered proGuide-based system described here satisfies many of the essential parameters described above while requiring no genetic engineering or mutation of the cell’s genome. We suggest that the proGuide system provides a powerful tool to take the existing rich understanding of sequential gene expression from single-cell RNA sequencing (RNA-seq) datasets and begin to use it for engineering autonomous sequential gene activation programs to drive the differentiation of stem cells.

## RESULTS

### RNA Pol III terminator signals inactivate proGuides without the need for cis-acting ribozymes

**Fig. 1.**
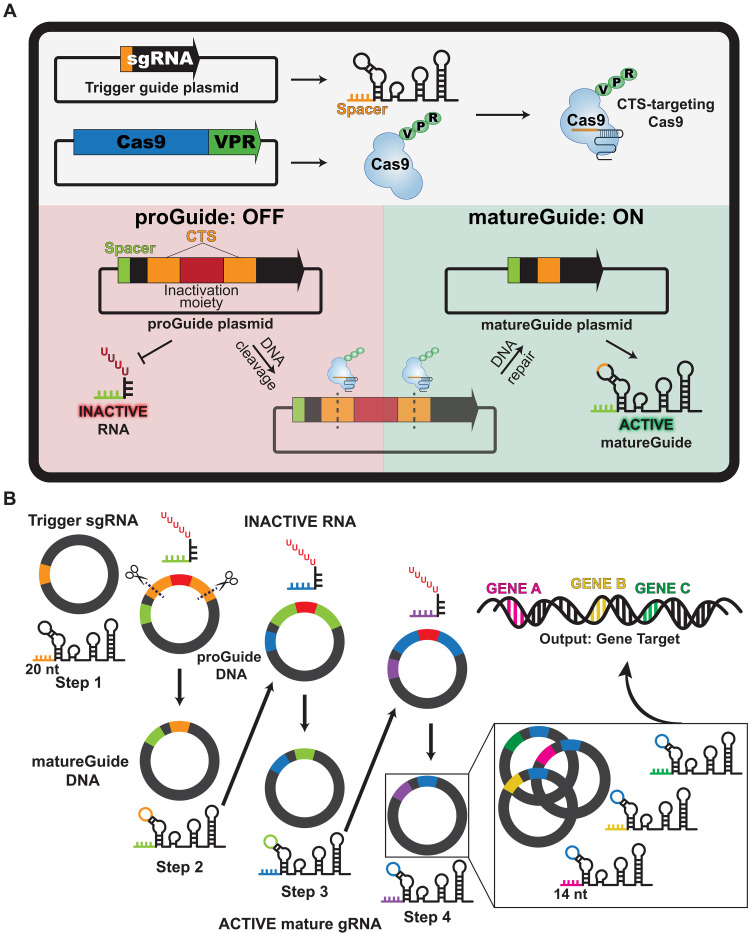
The proGuide is a conditional CRISPR sgRNA. (**A**) Schematic illustrating the conversion of an inactive proGuide to an active matureGuide. (Top) Plasmid DNA for Cas9-VPR expression and a trigger sgRNA plasmid encode the components, which when transfected into cells, target Cas9 activity to the CTS corresponding to the spacer (red). (Bottom left) In the absence of a trigger, an inactivation moiety embedded in the proGuide prevents activity as a guide RNA. (Bottom right) In the presence of a trigger guide RNA specific to the CTS (red), the inactivation moiety is excised from the proGuide plasmid DNA, resulting in transcription of an active matureGuide RNA. (**B**) Schematic illustrating progression of a proGuide cascade encoded in plasmid DNA. Color elements for steps 1 and 2 follow the rubric from (A). Steps 3 and 4 add new spacer and CTS for each step, and step 4 includes multiple proGuides with the same CTS (blue) and different 14-nt spacers target three genes for CRISPRa.

**Fig. 2.**
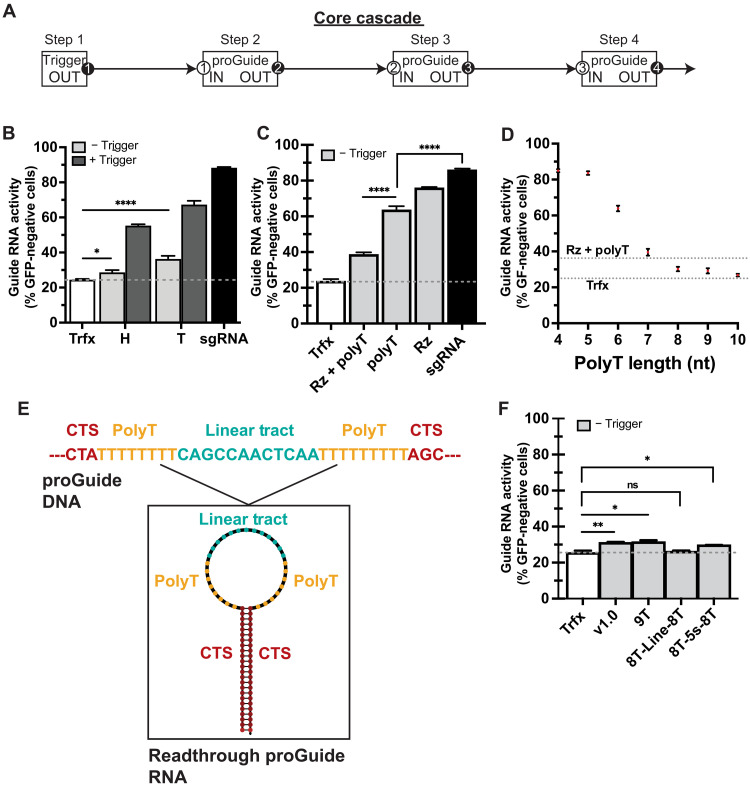
Inactivation of proGuides by RNA Pol III transcriptional termination sequences. (**A**) Schematic representation of a cascade of proGuide plasmids, wherein the spacer sequence functions as an output activity (black oval) of one guide RNA and corresponds to the CTS (white oval) as the sensing input in a downstream proGuide DNA. (**B** to **D**) Reduction of GFP-positive HEK293T cells harboring a genomic EGFP expression cassette was determined by flow cytometry 72 hours after transient transfection of guide RNA plasmids and a Cas9 expression plasmid. Control transfections either lacked an EGFP targeting guide RNA (Trfx; white bars, dotted line) or included an sgRNA targeting EGFP (sgRNA; black bars). (B) First-generation proGuides displayed activity in the absence of a trigger RNA (light gray bars) when inactivation sequences were positioned in the tetraloop (T) region. ProGuides exhibited incomplete conversion to an active state when located in the hairpin (H) region. (C) Inactivation with only the ribozyme (Rz), only the 6-nt polyT tract (PolyT) or both (Rz + PolyT) all displayed leakiness. (D) Increasing the length of the polyT tract reduced leakiness of EGFP proGuides below that of the first-generation inactivation sequences (Rz + PolyT dotted line). (**E**) Schematic of the DNA sequence (top) encoding a dual polyT tract and predicted RNA secondary structure (bottom). (**F**) Absence of detectable leak from proGuides harboring the dual polyT tract depicted in (E). All data represent mean of biological triplicates ± SD. Statistical significance is denoted as follows: *P < 0.05, **P < 0.01, and ****P < 0.0001; ns indicates *P* > 0.05 (unpaired *t* test). SD values smaller than the figure symbol were omitted for clarity.

The proGuide was considered for its ability to function as the molecular gate for construction of genetic circuits to program stepwise activation of endogenous genes in mammalian cells. The proGuide differs from a single guide RNA (sgRNA) by requiring a Cas9-mediated editing event to remove an inactivating sequence from its DNA, which converts it to an active, matureGuide encoding DNA (Fig. 1). By matching the sequence encoded by the 20–nucleotide (nt) spacer region from one guide RNA (Fig. 2A, black circles) to the 23-bp Cas9 target site (CTS) embedded within another proGuide’s DNA (Fig. 2A, white circles), a cascade of several proGuides can be activated in a sequential manner. As a cascading system, inefficiencies at each upstream step affect all downstream steps such that a relatively low level of nonspecific activity in the absence of a trigger (i.e., leak) is compounded at each step disrupting the syntax of the system. Ideally, there would be no leak in each individual proGuide in the absence of a trigger. In terms of level of activity in the presence of a trigger, we suggest that an optimal proGuide should be able to be converted to display the same activity in cells as an sgRNA does. Using these performance targets, previously published proGuides (v1.0) (*23*) with insertions in the tetraloop or hairpin 1 location were notably less effective than an sgRNA for Cas9-mediated disruption of a genomic enhanced green fluorescent protein (EGFP) transgene in human embryonic kidney (HEK) 293T cells (Fig. 2B and fig. S1). In addition, the tetraloop variant displayed significant residual leak in the absence of a trigger guide RNA (Fig. 2B).

To elucidate the causes of deficiencies and to improve system performance, proGuide leak and activity were tested independently. Initial proGuide designs incorporated redundant mechanisms in the DNA sequence designed to interfere with the activity of the encoded guide RNA (*23*). A cis-cleaving hammerhead ribozyme sequence was included to cleave the RNA before binding to Cas9, and a polyT tract of six consecutive thymidine residues was designed to terminate RNA transcription preventing synthesis of a full-length functional guide RNA (fig. S2). The impact of each inactivation moiety was tested by constructing proGuide variants having either a hammerhead ribozyme or the polyT tract alone within the loop structures of the tetraloop or hairpin 1. The polyT tract was more effective than the ribozyme in mitigating unwanted guide RNA activity when placed in either the tetraloop or the hairpin locations (Fig. 2C and fig. S3). Conversely, using a full-length hammerhead ribozyme with purported enhanced RNA nucleolytic activities as the inactivation moiety unexpectedly exacerbated proGuide leak (fig. S4). Given that the enhanced activities of the full-length ribozyme are dependent on RNA folding (*24*) and placement of the ribozyme within the context of a proGuide may obstruct this folding, we concluded that it would be challenging to design a proGuide devoid of leak using ribozyme-based inactivation.

Several previous observations supported focusing on the polyT tract for development of more effective inactivation moieties. Inactivation by the polyT tract relies on its ability to terminate elongation of RNA polymerase III (RNA Pol III), precluding transcription of the downstream guide RNA sequence, which is necessary for Cas9 activities (*25*). Several known parameters, including the number of consecutive thymidines, the promoter initiating transcription, and the nucleotide sequence upstream of the polyT tract, have been shown to control the extent of termination (*26*, *27*). When incorporated as the sole inactivation moiety in proGuides, increasing the number of consecutive thymidines in a polyT tract progressively reduced leak, with the most substantial mitigation in leak observed when lengthening the polyT tract from four to eight thymidines (Fig. 2D). Addition of a 9th or 10th consecutive thymidine provided minimal additional reduction in activity (Fig. 2D and fig. S5). DNA encoding the RNA fragment expected from termination by the polyT tract but lacking guide sequences downstream of the polyT tract failed to disrupt EGFP in cells (fig. S6), indicating that the residual leak resulted from transcriptional readthrough rather than a low-level activity of the RNA fragment terminated at the polyT tract.

To eliminate the residual leak, a dual polyT tract strategy similar to those observed in some tRNA genes was tested (*26*). Given that the RNA secondary structure preceding the terminator has been suggested to enhance termination (*27*), two different DNA sequences were evaluated for the RNA structures that they encoded. One sequence encoded a 5*S* ribosomal RNA structure that was previously shown to stimulate RNA Pol III termination in mammalian cells, whereas the other encoded an RNA motif predicted to confer a linear conformation. Both structures were flanked between two terminator tracts (Fig. 2E) (*27*). Unexpectedly, configuration of two 8T tracts separated by the linear tract resulted in the lowest amount of leak, which was comparable to the control condition without any guide RNA (Fig. 2F). Given that these results were from tetraloop-modified proGuides, which had previously displayed greater leak and higher conversion activity (Fig. 2B) (*23*), this inactivation sequence was used as the foundation for further development of the proGuide as an effective molecular gate.

### Orientation of the CTS sequences in the proGuide DNA template determines DNA repair and efficacy of the matureGuide

The efficacy of a proGuide in mammalian cells depends on both its conversion rate to a functional matureGuide and the activity of the resulting matureGuide RNA. Ideally, a matureGuide would exhibit the same level of activity as an sgRNA; however, previously published proGuides frequently displayed activity significantly lower than that of an sgRNA (Fig. 2B) (*23*). Although the Cas9 guide RNA can accommodate sequence additions in the tetraloop and hairpin 1 loops, even minor modifications to the guide RNA backbone can reduce its activity (*25*, *28*), suggesting that configuration of the CTS sequences could affect guide efficacy.

**Fig. 3.**
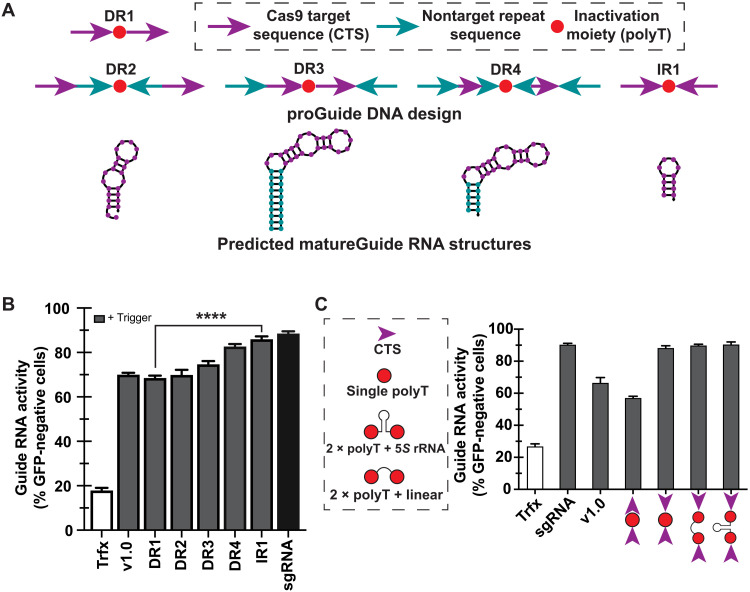
Effects of the orientation of CTS repeats on conversion of proGuides to active guide RNA. (**A**) Schematic of the arrangement of different proGuide components in a DNA sequence (top) and predicted RNA sequence structures (bottom) following a perfect NHEJ repair–mediated nested deletion between the two Cas9 cut sites. (**B**) Transient transfections (as described in Fig. 2) with proGuide expression plasmids depicted in (A). (**C**) Conversion of IR1 configuration to an active guide RNA state was not significantly changed by the termination sequences (schematic on left). All data represent the mean of biological triplicates ± SD. **** indicated *P* < 0.0001 (unpaired *t* test). rRNA, ribosomal RNA.

To determine whether the proGuide architecture could affect the activity of matureGuides, different proGuide configurations were assessed for activity in response to a trigger. The CTS sequences were identical for all proGuide configurations to eliminate potential confounding effects of variations in EGFP disruption caused by the activity of the trigger sgRNA on the CTS. Similarly, the inactivating moiety was also kept constant in the five proGuide configurations, which differed by orientation of the CTS as a direct repeat (DR) versus inverted repeat (IR), by the presence of additional DNA repeats nested between the two CTS, and by the presence of additional DNA repeats flanking the two CTS (Fig. 3A). Secondary structures of the RNA sequence at the end of the tetraloop were predicted assuming perfect nonhomologous end joining (NHEJ) repair of the DNA (Fig. 3A), and they illustrated the base pairing of nucleotides encoded by IRs forming additions to the stem structure in the tetraloop region (Fig. 3A).

In cells, these engineered configurations displayed a range of matureGuide activities (Fig. 3B and fig. S7). Although DR1 and DR2 variants had a simpler design relative to previously published proGuides (v1.0), these new variations did not improve editing activity in response to a trigger (Fig. 3B). Placing DNA repeat sequences to flank the CTS in DR3 and DR4 increased matureGuide RNA activity in response to a trigger, suggesting that the stem structure resulting from the repeat sequence supported functionality of the guide RNA and may be necessary for high matureGuide RNA activity (Fig. 3, A and B). The shorter stem structure of DR4 resulted in higher matureGuide RNA activity than the longer stem from DR3. Consistent with the role of the stem structure, the IR1 configuration generated the highest matureGuide RNA activity. Moreover, the predicted post-NHEJ structure for the IR1 configuration had one of the most stable hairpin structures, which would support the tetraloop structure of the guide RNA. Note that perfect NHEJ repair between two CTS configured in a so-called protospacer adjacent motif (PAM)–out orientation of IR1 results in 12-bp IR, consisting of the 3-bp PAM sequence and PAM-proximal 3 bp from each CTS. Replacing the inactivation moiety with more effective ones (Fig. 2, D and E) indicated that effectiveness of the IR orientation was not dependent on a specific sequence between repeats (Fig. 3C). The superiority of IR orientation was also consistent in proGuides with other CTS sequences (fig. S8), suggesting that the effect was not caused by altering the nuclease activity of Cas9 at individual CTS sequences. Since previous research also demonstrated increased guide RNA activity from stabilization of the tetraloop (*28*), we suggest that the 6-bp supporting stem structure from IR1 configurations likely stabilized the hairpin structure of the matureGuide tetraloop, improving efficacy of matureGuides.

**Fig. 4.**
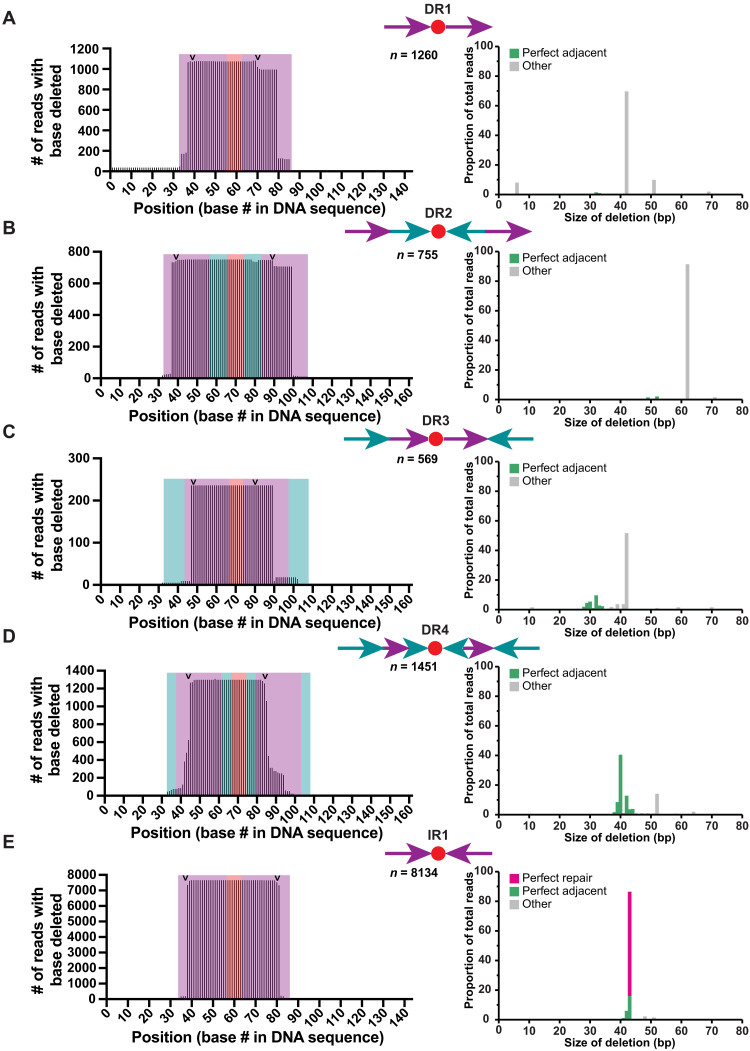
Effects of orientation of CTS repeats on the RNA sequence of converted matureGuides. (**A** to **E**) DNA sequencing results of reverse transcribed guide RNA from HEK293T cells transiently transfected with proGuides containing CTS configurations depicted in Fig. 3A. Sequences were analyzed and stratified on the basis of RNA transcript size and mapped to proGuide reference sequences. The frequency of deletions at each base in the proGuide (left graph) is depicted with the expected Cas9 cleavage sites assuming perfect NHEJ repair (arrows). Shading corresponds to regions of the CTS (purple), nontarget repeat sequence (teal), and polyT inactivation moiety (red). The distribution of deletion sizes for each CTS variant (right graph) is depicted with perfect repair between the Cas9 cut site (pink), near-perfect repair within 3 bp at either end of the Cas9 cleavage site (green), or other deletion sizes (gray). proGuide DNA containing CTSs oriented as IRs (IR1) displays higher frequency of perfect repair than DR sequences. Data represent all sequences with at least two reads in a sample.

To determine the effect of DNA repair on the efficiency of proGuide conversion to matureGuides, we began with an RNA-seq approach. Briefly, cells were transfected with proGuide, Cas9, and trigger sgRNA plasmids, and RNA was isolated and amplified for sequencing of RNA molecules having the 3′ guide RNA sequence downstream of the polyT tract. Cas9-mediated changes to proGuide plasmids were characterized by mapping sequencing reads onto the proGuide DNA reference sequence. The previously published proGuide architecture generated a diverse set of RNA sizes in cells (fig. S9), including 35% of sequences aligning to the 254-bp proGuide sequencing caused by readthrough transcription and failure of ribozyme cleavage of the proGuide RNA transcript. Unexpectedly, 166-bp RNA, corresponding to the size expected from perfect NHEJ repair, made up only 1.5% of transcripts (fig. S9A). In contrast, proGuides harboring CTS in an IR orientation produced RNA sequences corresponding to the perfect NHEJ repair between the two CTS (fig. S9B). Comparison of RNA produced from conversion of DR1-4, IR1 proGuides (Fig. 4, A to E) revealed that none of the DR configurations generated RNA corresponding to predominantly perfect repair between CTS (tables S2 to S7). Instead, most frequent outcomes had additional deletion of CTS sequences, both upstream and downstream of the Cas9 cut site. The DR4 configuration produced the most variable outcomes with the predominant 40-bp loss occurring in only 40% of deletions shown in Fig. 4D. Outcomes from conversion of DR1 were notable, because it was the only configuration to produce a substantial frequency of nondeletion changes, which included 5% of mapped reads corresponding to a perfect inversion of the sequence between the two CTS (table S2). By contrast to the DR configurations, the large majority (63%) of outcomes from IR1 corresponded to a perfect deletion between the two CTS (Fig. 4E and table S2). Regardless of CTS configuration, less than 1% of RNA sequences corresponded to readthrough guide RNA, which was consistent with very low levels of leak from this proGuide in cells (Fig. 2, D and F). The frequency of RNA corresponding to either a perfect repair or insertion/deletion of 1 bp between the two CTS from IR1 was consistent with a structurally permissive and thus highly effective form of matureGuide RNA displaying high activity in cells. The markedly different frequencies of RNA sequences produced by proGuides with the same CTS sequences, but in different orientations, suggest that the DNA repair process is affected by CTS orientation. One possible explanation is that perfect NHEJ repair of CTS in a DR orientation will form an intact single CTS in the plasmid, which would provide a substrate for additional Cas9 cutting.

### High-efficiency CTS sequences are required for multistep cascades of proGuides

**Fig. 5.**
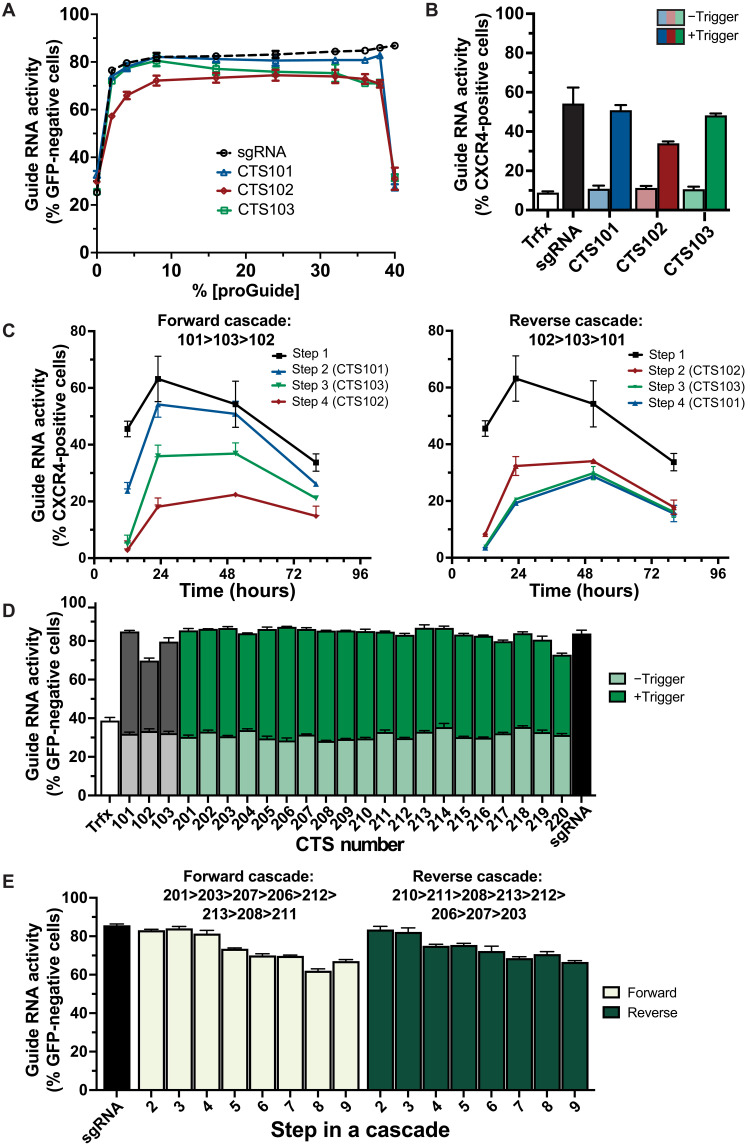
Sequence composition of CTS affects efficiency of proGuide conversion. (**A**) The EGFP disruption activity of different ratios of proGuide: trigger plasmid DNA was determined by flow cytometry 72 hours after transient transfection (similar to Fig. 2). Three proGuides and their matched trigger plasmids used different CTS (CTS101, CTS102, and CTS103). (**B**) Activation of the endogenous CXCR4 gene determined by cell surface protein expression was determined by flow cytometry 48 hours after transient transfection of a proGuide (CTS101, CTS102, and CTS103) with a 14-nt spacer targeting the CXCR4 promoter, a trigger plasmid, and a Cas9-VPR expression plasmid. Transfections lacking a guide RNA and with an sgRNA with the 14-nt CXCR4 spacer were used as negative/positive controls. (**C**) Two proGuide cascades constructed in opposing directions using spacers and CTS sequences corresponding to CTS101, CTS102, and CTS103 (fig. S10). Cascade plasmids were transiently transfected into HEK293T cells with a Cas9-VPR expression plasmid and assessed by flow cytometry for surface protein expression of CXCR4. (**D**) Activity of proGuides (+/− trigger) harboring 20 engineered CTS sequences determined by EGFP disruption, as described in Fig. 2 and fig. S1. (**E**) Two proGuide cascades in opposing directions using engineered CTS sequences (D) in the sequential order indicated above the graph. All data represent the mean of biological triplicates ± SD. SD values smaller than the length of the figure symbol were excluded for clarity.

Whereas experiments evaluating the inactivation moiety (Fig. 2) and the proGuide architecture (Figs. 3 and 4) kept the CTS sequence constant to control for effects of Cas9 nuclease activity, we anticipated that CTS sequences would require optimization to build efficient multi-proGuide cascades. We considered that one potential limitation could come from competition for common resources (e.g., Cas9 protein, number of transfected plasmids, RNA Pol III, etc.) and maintaining efficiency of each individual proGuide step when multiple proGuide plasmids and matureGuide RNA are present in individual cells. To determine the efficacious dose range of a proGuide, EGFP disruption was measured in cells transfected with different proGuide:trigger sgRNA ratios, ranging from 20:1 to 0.05:1 (Fig. 5A). In general, across the titration, all three proGuides exhibited ubiquitous activity over a wide dose range. However, compared to CTS101, which exhibited sgRNA-like activity across the titration, CTS102 and CTS103 exhibited deficiencies. Regardless of the relative amount of the CTS101-containing proGuide, it caused GFP disruption similarly to the sgRNA control, and it was notably effective even at the lowest concentrations representing 2% of the total plasmid DNA delivered to cells. CTS102 generated the lowest levels of EGFP disruption at all concentrations (Fig. 5A), suggesting a deficiency in the matureGuide RNA activity independent of the CTS102-specific trigger sgRNA. In contrast, CTS103 displayed sgRNA-like activity only at low proGuide concentrations but reduced activity when the ratio of trigger RNA was increased, suggesting a deficiency in conversion by the CTS103-specific trigger sgRNA (Fig. 5A).

To evaluate effects of CTS sequences on proGuides for transcriptional activation of endogenous genes, a nuclease active Cas9-VP64-p64-Rta (Cas9-VPR) fusion protein was used for both conversion of proGuides to matureGuides and for CRISPR activation (CRISPRa) of genes. The two activities can be separately targeted by using 20-nt spacers for Cas9 nuclease activity and 14-nt spacers for DNA binding to promoters of target genes (*29*). The C-X-C chemokine receptor type 4 (CXCR4) cell surface protein was used as an endogenous reporter gene due to the simplicity of detection by flow cytometry and previous reports showing its activation with dCas9-VPR, an endonuclease-inactive Cas9 fused to a tripartite transcriptional activator composed of VP64, p65, and Rta (*29*). Several 14-nt spacer sequences were evaluated for activation of CXCR4 expression in HEK293T cells (fig. S10), and the most active spacer was cloned into proGuide plasmids. Plasmids encoding a proGuide with a 14-nt spacer targeted to *CXCR4*, a trigger sgRNA, and Cas9-VPR expression were cotransfected into HEK293T cells, and the frequency of cells displaying CXCR4 expression was measured after 48 hours by flow cytometry (Fig. 5B). Given that CXCR4 expression was dependent on the inclusion of a trigger sgRNA plasmid, these results indicate that Cas9-VPR was able to catalyze both the conversion of a proGuide to a matureGuide and the activation of *CXCR4* transcription from its endogenous gene. The different frequencies of *CXCR4* activation from proGuides with different CTSs suggest that the activation of an endogenous gene may be more sensitive than the disruption of EGFP to conversion efficiency and efficacy of matureGuides.

Effects of CTS sequences with variable activities were tested by assembling multistep proGuide cascades, which required Cas9 nuclease for stepwise progression and required VPR-stimulated transcriptional activation for endogenous gene expression (fig. S11). Two cascades were generated from proGuide plasmids, each containing spacers designed in one step to target the CTS of the next step’s proGuide in either forward or reverse directions. (Fig. 5C and fig. S11). Changes to the cell surface expression of CXCR4 over time indicated that proGuides containing CTS102 reduced activation substantially at the step requiring CTS102 and also at all downstream steps (Fig. 5C). CTS103 also caused reduced activation in both cascades, and because of the successive nature of cascades, this resulted in a low-level of activation of steps 2, 3, and 4 in the reverse cascade. By contrast, steps requiring proGuides with CTS101 displayed the least amount of lost activity compared to previous steps. The inefficiency of CTS102 combined with the high efficiency of CTS101 in the following step of the reverse cascade resulted in a timing defect, whereby steps 3 and 4 failed to display the expected syntactic separation. Together, these results identified possible modes of failure for cascades of proGuides and suggested a need for optimized CTS sequences to generate efficient and effective proGuides.

To generate CTS sequences that would support efficient cascades of proGuides, a virtual library of 2 million random 23-bp CTS sequences was generated and filtered to remove sequences with features (e.g., guanine-cytosine (GC) content, PAM-distal guanine content, and PAM-proximal thymidine content), previously shown to impair Cas9 activity (fig. S12) (*30*–*33*). To minimize potential Cas9 nuclease activity on the human genome and other commonly used animal genomes, CTS sequences were filtered to ensure at least seven mismatches with any sequence in genome assemblies from human, mouse, cow, pig, tuna, and chicken. Restriction through filters resulted in more than 50,000 possible CTS sequences, all predicted to direct no on- or off-target Cas9 activity in the genome for the selected organisms (fig. S12).

A set of 96 of these CTSs was randomly selected for empirical testing in human cells. The selected CTS and the matching spacer sequence were cloned into a plasmid enabling the expression of an EGFP-targeting proGuide and its trigger sgRNA from a single plasmid. Loss of green fluorescent protein (GFP) fluorescence 48 hours posttransfection showed a range of CTS functionality, including several as active as CTS101 (Fig. 5D and fig. S13). The top 20 CTS sequences were cloned into proGuide plasmids, their matching spacers were cloned into separate trigger sgRNA expression plasmids, and each combination’s ability to disrupt EGFP in cells was compared to CTS101 to CTS103 proGuides (Fig. 5D). As expected, 20 proGuides containing CTS201 to CTS220 sequences exhibited negligible leak and high matureGuide activity when delivered with its trigger sgRNA (Fig. 5D). Next, proGuide plasmids containing CTS201-220 were constructed with the 14-nt spacer for transcriptional activation of CXCR4 and evaluated for activity with Cas9-VPR. In this CRISPRa activity assay, all proGuides displayed negligible leak and minimal variability in matureGuide activity (fig. S14). Several CTSs (CTS201, 203, 207, 206, 212, 213, 208, and 211) that performed well in both the EGFP disruption and CXCR4 activation assays were chosen for evaluation in proGuide cascades using only these top-performing second-generation CTS (CTS2XX) sequences.

Initial tests of the new CTS in proGuide cascades measured the ability of an EGFP-targeting proGuide placed at individual steps in nine-step cascades. Each nine-step core cascade was generated by inserting 20-nt spacer sequences into proGuide base plasmids such that each proGuide would target the CTS of the next proGuide in either forward or reverse cascades depicted atop (Fig. 5E). Each transfection mix contained proGuide plasmids constituting a cascade, a Cas9-VPR expression plasmid, and one EGFP-targeting proGuide that had the CTS programming activation at only one step from 2 to 9. Total EGFP disruption decreased as steps progressed in both forward and reverse cascades (Fig. 5E). Notably, the stepwise decrease in activity did not correspond to specific CTS, suggesting a high degree of modularity with the chosen proGuide CTS and spacer components.

### proGuide cascades enable sequential and multiplexed transcriptional activation of endogenous genes

**Fig. 6.**
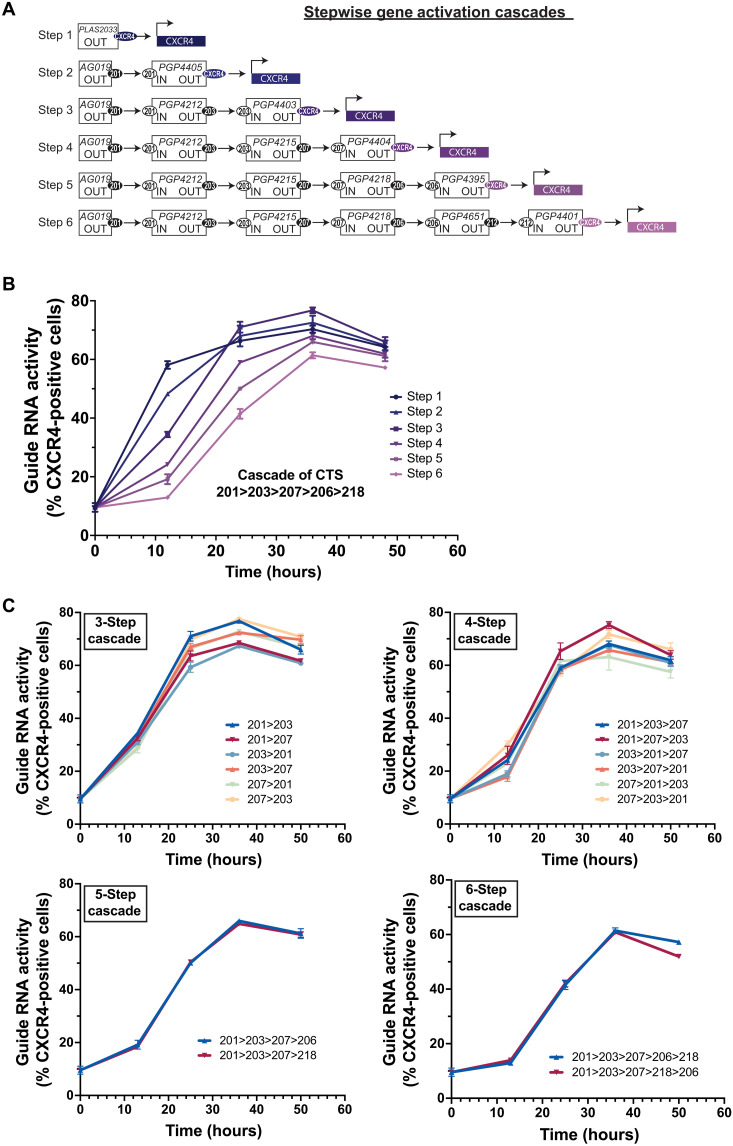
Control over kinetics of endogenous gene activation by transient transfection of proGuide plasmids. (**A**) Schematic depicting plasmid DNA composition of proGuide cascades used in (B), as described in fig. S11. (**B**) CXCR4 surface protein expression was measured by flow cytometry at 12-hour intervals after transient transfection of HEK293T cells with Cas9-VPR and plasmid mixes depicted in (A). (**C**) The order of the CTS used in cascades of proGuides was iterated on by generating new proGuide plasmids for stepwise progression to activation of CXCR4 transcriptional activation by Cas9-VPR. Each graph shows CXCR4 surface protein expression for cascades designed to activate CXCR4 at the indicated step number. All data represent the mean of biological triplicates ± SD. SD values smaller than the length of the figure symbol were excluded for clarity.

To determine whether a cascade of proGuides could promote stepwise activation of endogenous genes, a core cascade of five proGuides was combined with a proGuide targeting *CXCR4* for transcriptional activation at one step according to the identity of its CTS (Fig. 6A). Similar to proGuide cascades used for disruption of EGFP (Fig. 5E), the CXCR4 activation cascades were efficient in terms of the percentage of cells that completed a six-step cascade and activated the last step in the cascade (Fig. 6B). Comparison of cascades at different time points showed that placement of the CXCR4 proGuide at different steps resulted in the expected sequential activation of cell surface expression, with a lag period of up to 6 hours between steps (Fig. 6B). Experiments examining all combinations of cascades with proGuides containing CTS 201, 203, and 207 for steps 2, 3, and 4 indicated that these different proGuides functioned similarly within cascades (Fig. 6C), supporting the modularity of proGuides as a molecular gate for the activation of endogenous genes by CRISPRa. In addition, 10-step cascades were evaluated to test the upper limit to the number of steps that could be programmed with the next-generation proGuides (fig. S15). The kinetics of CXCR4 activation in these 10-step cascades displayed sequentially separated activation up until step 8, at which point the overall activity remained but syntactic separation between individual steps was lost (fig. S15).

**Fig. 7.**
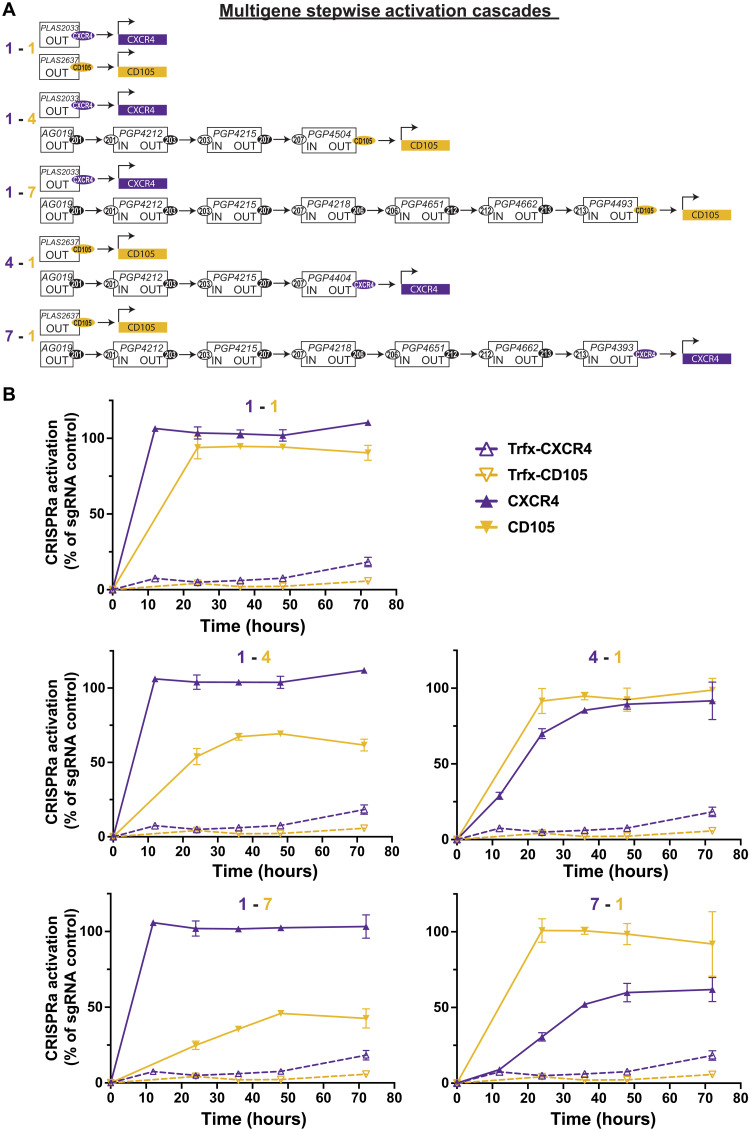
Syntactic activation of CD105 and CXCR4 genes using cascades of proGuides. (**A**) Schematic depicting plasmid DNA composition of proGuide cascades used in (B). (**B**) CXCR4 and CD105 surface protein expression was measured by flow cytometry at 12-hour intervals after transient transfection of HEK293T cells with Cas9-VPR and plasmid mixes depicted in (A). Graphs show the percentage of cells positive for CXCR4/CD105 relative to the number of positive cells observed in sgRNA-positive control transfections at each time point. Note that since the sgRNA-positive control did not display CD105 positivity at 12 hours, normalized data are not shown for CD105 at that time point. All data represent the mean of biological triplicates ± SD. SD values smaller than the length of the figure symbol were excluded for clarity.

To potentially direct cellular differentiation through steps in a lineage, proGuide cascades would need to activate multiple endogenous genes in a preprogrammed syntactic manner. We began to evaluate such capabilities to program the activation of CXCR4 and Endoglin (CD105) each at different steps in HEK293T cells. Briefly, a seven-step core cascade was used for all conditions, and proGuides activating the endogenous genes were included for activation in steps 1, 4, and 7 (Fig. 7A). Kinetics of activation were examined by flow cytometry every 12 hours after DNA delivery. In transfections where both genes were programmed for activation at step 1 via sgRNA, cell surface expression of CD105 occurred ~12 hours after CXCR4, suggesting a delay in CD105 independent of any control programmed by proGuides cascades (Fig. 7B). Switching the order of CXCR4 and CD105 in step 1 versus 4 or step 1 versus 7 changed the order in which the surface expression of the two gene products were detected on individual cells (Fig. 7B). Consistent with cascades activating stepwise expression of a single (Fig. 6) gene, placing gene activation proGuides at a later step in a cascade delayed expression of one gene product relative to another placed at an earlier activation step.

**Fig. 8.**
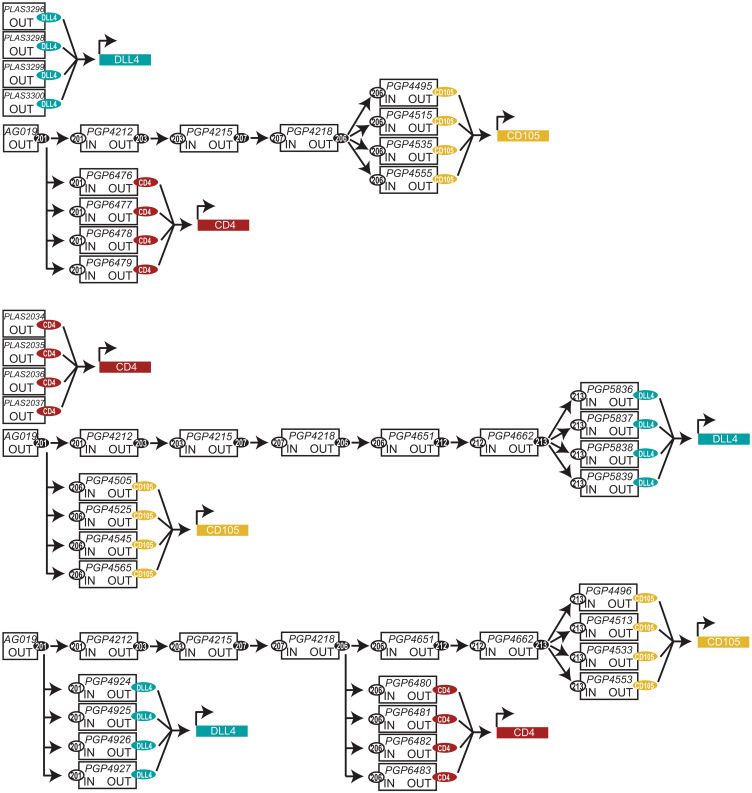
Pool of proGuide plasmids developed for use in iPSCs. Schematic illustrates proGuide plasmid arrangement in three different cascades, each designed to activate different permutations of DLL4, CD4, and CD105 at step 1, 2, 5, or 7. The schematic follows the format described in fig. S11. Note that four guide RNAs are used to target the CRISPRa activity on each endogenous gene with binding of four different spacer sequences arranged on the gene’s promoter region.

Cascades of proGuides were tested in human iPSCs to provide a better evaluation of their potential for programming cell differentiation in a stem cell system capable of making any somatic human cell type. Several parameters were considered for designing this test of the system’s capabilities. Flow cytometry was chosen for the main assay because it provided simultaneous quantitative measurements of multiple proteins in individual cells, and it could be used to rapidly compare many experimental conditions. Three target genes were chosen because they each encode a cell surface marker protein (CD4, DLL4, and CD105) expressed at low or undetectable levels in iPSCs and were stimulated by CRISPRa (fig. S16). Given previously described sensitivity of human iPSCs to exogenous DNA (*34*), it was important that the evaluation was performed with cascades that represented a level of complexity commensurate with those that would be needed to activate several TFs at multiple steps. Therefore, we increased the number of plasmids nucleofected into iPSCs (19 minimum) by using a pool of four guide RNA to target activation of each of the cell surface proteins (Fig. 8). The pools of four guide RNA were made on the basis of previously published guidelines (*35*), and such that the same CTS triggered all of the four pooled proGuides for a single-cell surface protein (Fig. 8). This strategy for increasing the complexity of otherwise simple cascades provided the benefit of also achieving CRISPRa activity without having to screen to identify an effective individual guide RNA for each gene. sgRNA and proGuides stimulated similar distributions of levels of cell surface markers on individual cells although the frequency of expressing cells diminished as expected during progression through proGuide steps (fig. S16). These observations are consistent with the concentration of the proGuide and trigger guide being relatively less important (Fig. 3A) compared to the importance of the spacer sequence(s) used to target Cas9-VPR to the promoter of the target gene (*35*–*37*).

**Fig. 9.**
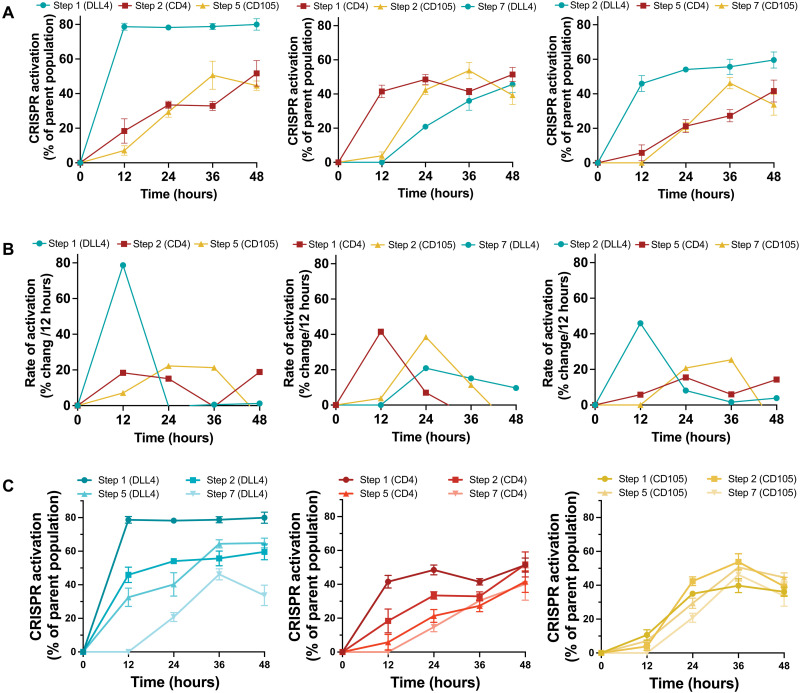
Syntactic activation of several genes in iPSCs using cascades of proGuides. (**A**) The expression of cell surface marker proteins, DLL4, CD4, and CD105, in human iPSCs after nucleofection of one of the proGuide cascades shown in Fig. 8. Protein expression was measured every 12 hours by flow cytometry, and the gating strategy described in fig. S17 was used to determine the percentage of positive cells from the parental population [i.e., those expressing the indicated marker from the pool of cells that expressed the marker(s) upstream in the cascades]. Each graph corresponds to one of the cascades shown in Fig. 8. (**B**) The rate of activation of each gene was determined from the frequencies in (A) and represented as the percent increase per 12 hours. (**C**) Relative activation of surface marker proteins, DLL4, CD4, and CD105, in iPSCs programmed at steps 1, 2, 5, and 7. Each graph shows data from cells nucleofected with different proGuide cascades. All data represent the mean of biological triplicates ± SD. SD values smaller than the length of the figure symbol were excluded for clarity.

The analysis of the effects of proGuide cascades in iPSCs was focused on the control over the syntax by which the three genes were activated relative to one another. A parental gating strategy was used for flow cytometry analysis to determine the frequency by which a later step occurs in cells that have completed an earlier step (fig. S17). Note that similar to activation CXCR4 and CD105 in HEK293 cells (Fig. 6), gene products were detected as cell surface markers at different rates after nucleofection of CRISPRa plasmids into iPSCs, with DLL4 expression occurring sooner than CD4 and CD105 (Fig. 9). Comparing the timing of different markers among the three different cascades, the relative order of initial cell surface expression was determined by the step used to program the CRISPRa activation of the gene (Fig. 9A). For example, surface expression of DLL4 was detected before other genes when programmed at step 1 and after the other genes when programmed at step 7. Comparison of the rates of activation indicated that earlier steps (step 1 and step 2) produced a more rapid increase among the population of cells than the later steps (step 5 and step 7) (Fig. 9B), which is consistent with a lower frequency of positive cells in later steps (fig. S16). As expected, comparisons focused on a single gene at different steps showed that it took more time to be activated from later steps than earlier steps (Fig. 9C). Together, these data demonstrate that cascades can establish diverse sequential gene activation programs in stem cells, enabling the activation of multiple genes at different times relative to one another within individual cells. Given the flexibility of Cas9-VPR for activation of virtually any gene in the genome, complex programs of sequential gene activation processes are likely possible.

## DISCUSSION

Since the discovery of the ability of myogenic differentiation factor 1 (MyoD) to program fibroblasts to differentiate into skeletal muscle cells (*4*), substantial efforts have identified key TF genes and cell types amenable for programming with exogenous, transgenic expression of TFs in cells (*2*). Subsequently, the development of genetic engineering and synthetic biology tools has allowed more sophisticated programming using molecular gates that can elicit cellular effects depending on the presence or absence of a trigger (*38*, *39*). This work enhances cell programming by creating a system that efficiently activates at least seven genes in sequence, with each activation triggered by the successful completion of the previous step. The number of steps, the capacity to activate any gene in the genome, and the efficiency of stepwise progression are each consequential advances in mammalian synthetic biology.

The focus of the current study was to transform the proGuide into an effective and modular molecular gate for programming activation of endogenous genes. However, several findings from this endeavor could have substantial and broader implications independent of proGuides. Notably, simple inactivating moieties composed of polyT tracts decisively prevented RNA Pol III synthesis of functional sgRNA transcripts. We propose that the polyT tract is a more effective inactivation method than ribozymes because it blocks RNA synthesis directly, rather than relying on a self-destructive nucleolytic reaction that depends on proper ribozyme folding (*40*). Furthermore, manipulation of the length and number of polyT tracts enables an additional layer of modularity. The mechanism of polyT tract inactivation should apply to any RNA produced by RNA Pol III (*26*), assuming that the polyT termination tract precedes the functional component of the RNA. Hence, the polyT tract could inactivate microRNAs, small hairpin RNAs, ribozymes, and other RNA-guided enzymes, all of which typically use RNA Pol III for expression in mammalian cells. Conversion of such RNAs to an active state would depend on both the mechanism used to remove or disrupt the polyT tract and also the tolerance for extra nucleotides in the sequence that may remain after conversion. We suggest that the removal of polyT tracts via Cas9 deletion could provide conditionality to RNA Pol III transcripts analogous to how Lox-Stop-Lox cassettes have been used for RNA Pol II transcripts (*41*, *42*).

Analysis of guide RNA transcripts from plasmid DNA provides insight into the repair of extragenomic DNA following Cas9 cleavage in mammalian cells. In comparison to the abundance of research into the repair of genomic DNA following Cas9 cleavage (*43*–*45*), effects on plasmid DNA are relatively unknown. Plasmid DNA harboring CTS arranged in a DR configuration rarely resulted in an NHEJ repair process with a perfect nested deletion between cut sites. By comparison, repair of a genomically integrated copy of the DNA sequence was reported to cause nested deletion between sites in about one third of edited DNAs (*23*). Changing the configuration of the CTS from DR to IR in plasmid DNA markedly increased the frequency of perfect nested deletions. We suggest that the perfect repair with IR configurations is consistent with frequent monomolecular (i.e., the ends of one plasmid DNA) repair process and infrequent interactions between two plasmid DNA molecules. In addition, the lack of perfect repair from DR configurations could be explained by a perfect repair between the CTS resulting in a reformation of an intact CTS, which can be cut again by Cas9. The predominant outcome from these secondary cuts appeared to be small deletions. An exception to this possibility was the frequent inversion of the complete DNA sequence nested between cut sites in DR4, which has an etiology that remains unclear to us. Together, these results suggest that repair of double-stranded breaks of plasmid DNA may be affected by different parameters than genomic DNA.

The proGuide-based cascades developed here have generated additional capabilities that could begin to be applied to programming cells. One distinguishing characteristic of these cascades from previously developed synthetic gene circuits is the autonomous nature of proGuide cascades. Once initiated by a trigger guide RNA, the cascade progresses without the need for conditional activation from the user via an external stimulus (e.g., small molecule, light, and temperature) (*15*, *20*, *46*–*48*) or via cellular activity (expression from conditionally active promoter and processing by a conditionally active enzyme) (*49*, *50*). Previous systems have been developed for autonomous progression, including one using an initial proGuide and another relying on the conversion of an inactive Cre-Lox system for guide RNA inactivation. Both systems could target the genome with various Cas9-mediated activities (*23*, *51*), similar to the approach presented here. However, their efficiency was extremely low, allowing completion of only one to two steps in cells (*23*, *51*). Other systems using base editors to make changes to the sequence of guide RNA were able to complete multiple steps, albeit in less than 10% of cells (*52*). In addition to the low efficiency, base editor systems are also limited, because they do not have a built-in capability for addressing endogenous genes in the mammalian genome. Instead, they require additional synthetic DNAs to be introduced into the cell for a functional gene expression output. By contrast, proGuides displayed high efficiency and syntactic activity for at least seven steps when delivered to mammalian cells as plasmid DNA, and they can be directed to turn on transcription of endogenous genes without requiring genomic insertion of heterologous DNA or mutation to the host cell genome. We suggest that these engineered capabilities give proGuide-based cascades unique advantages for the purpose of programming the differentiation of cells through sequential intermediate cell states.

Some characteristics of current proGuide cascades and their use in cells provide opportunities for further development of the system. Considering that the transition between cell states is thought to occur most effectively when it coincides with cell division (*53*), an ideal system might provide new genetic instruction sets (i.e., set of TFs to be expressed) coinciding with cell division. To reach this timing, the dwell time between steps of current proGuide cascades would need to at least double from one step every 6 hours to every 12 or 24 hours. Note that individual cells in populations progressed at different rates, and the goal would be to slow down the fastest conversions while maintaining efficiency. It would be worthwhile to evaluate several strategies for slowing progression, including replacing wild-type Cas9 with a modified Cas9 having slower or controllable endonucleolytic activity, such as high-fidelity Cas9 or photoactivable Cas9 (*15*, *54*, *55*). Mismatches between the spacer and CTS could also slow cutting without incurring an efficiency penalty caused by lowered binding of Cas9 to the CTS or lowered overall efficiency of cleavage. Empirically identifying mismatches that reduce the speed of conversion without substantially affecting efficiency of effective CTS sequences could generate considerably slower-progressing cascades. Slowing down progression through steps may also enable increasing the duration of the system to be more applicable to cell differentiation protocols that require timeframes of days to weeks. In addition, a substantial prolonging of the system could be achieved by increasing the maintenance of active plasmid DNA in dividing cells by addition of viral DNA retention elements (i.e., oriP-Epstein-Barr nuclear antigen 1) (*56*, *57*), use of miniaturized plasmid DNA forms (*58*, *59*), or mitigation of transcriptional silencing (*60*). Additional research would be required to elucidate the causes for loss of activity over time and to determine if they could be manipulated to extend the system.

Although the use of plasmids to encode the system likely contributes to its short duration, plasmids provide some substantial advantages. Integrating synthetic DNA elements into a mammalian genome requires weeks to months, but transfection of proGuide plasmids can be completed in a few days. They can be produced using simple and inexpensive recombinant DNA techniques that are routinely performed in most molecular biology laboratories. In addition, the generation of proGuide plasmids is scalable to make hundreds at once, and individual plasmids can be combined to make a variety of mixtures to formulate a large number of distinct cascades encoding the activation of different TFs and different steps.

The advances described in this study have converted proGuides from a proof-of-concept tool to a system that can be evaluated for programming mammalian cell differentiation. Given the complexity of cellular differentiation and the number of intermediate cell types identified by single-cell RNA-seq (*61*), seven addressable steps for programming cell state changes should be sufficient for deriving most cell types from a pluripotent stem cell starting point. The Cas9-VPR protein has been shown to display transcriptional activation functionality for a wide range of genes, making it possible to activate any gene in the genome with the existing system (*28*). Hence, the search space that can be addressed for permutations of TFs for effective differentiation of cells will most likely be limited by the ability to evaluate the effects on cells and not necessarily on the production of the proGuide cascade instruction sets. In summary, we propose that these engineered proGuides exhibit enhanced functionality across several parameters, including syntactic control of instructions, efficiency in progressing through multiple steps, minimal background activity (i.e., low leak), the ability to activate any gene in the genome, an increased number of programmable steps, and independence from genomic DNA breaks or the insertion of heterologous DNA elements.

## MATERIALS AND METHODS

### Plasmid DNA cloning

Standard molecular biology methods were used for engineering all plasmid DNAs used in this study (table S1). A base plasmid for expression of guide RNA was synthesized (GenScript) based on the sequence of the pSpgRNA plasmid (*62*) with modification to enable spacer sequences to be inserted by Golden Gate cloning with DNA oligos (Genewiz) as previously described (*63*). All plasmids encoding proGuide RNA variants were synthesized (GenScript) using the same backbone and spacer cloning strategy. Mix & Go competent DH5a (Zymo Research) was used for transformation of ligation reactions, and plasmid DNA was isolated from 50-ml overnight cultures with the ZymoPURE II Plasmid Midiprep Kit (Zymo Research). DNA sequences were verified by Oxford Nanopore DNA sequencing (Plasmidsaurus).

### Human cell lines and cell culture

The Lenti-X 293T (293-LX) cell line derived from HEK293T cells was obtained from Takara and used to generate an EGFP-expressing 293-LX cell line. 293-LX cultures were maintained in high-glucose Dulbecco’s modified Eagle’s medium (DMEM; Thermo Fisher Scientific) supplemented with 10% fetal bovine serum (FBS; GenClone) and penicillin/streptomycin (5 μg/ml; Thermo Fisher Scientific) and grown in ventilated flasks at 37°C in a 5% CO_2_ incubator before experiments. Cell cultures were routinely split at a 1:10 ratio every 3 days with dissociation agent TrypLE (Thermo Fisher Scientific).

Human iPSCs were obtained from American Type Culture Collection (HYR0103). Cells were maintained in Essential 8 (Thermo Fisher Scientific) with added supplement kit and penicillin/streptomycin (5 μg/ml; Thermo Fisher Scientific) with a daily change of media. Cells were grown on vessels coated with Vitronectin (Thermo Fisher Scientific) at a working concentration of 0.5 μg/cm^2^ in Dulbecco’s phosphate-buffered saline (Thermo Fisher Scientific). Once cultures reached 60 to 70% confluency, they were dissociated with Accutase (STEMCELL Technologies), resuspended in culture media supplemented with 10 μM ROCKi Y-27632 (Tocris), and allowed to grow for 24 hours postpassage, after which the media was exchanged for culture media (without ROCKi). Cells were maintained in ventilated flasks at 37°C in 5% CO_2_.

### EGFP–293-LX cell line production

293-LX cultures were maintained in high-glucose DMEM (Thermo Fisher Scientific) supplemented with 10% FBS (GenClone) and penicillin/streptomycin (5 μg/ml; Thermo Fisher Scientific). A day before transfection, 1.2 × 10^7^ cells were seeded into tissue culture–treated 150-cm^2^ ventilated flasks in 30 ml of culture medium. On transfection day, pseudotyped lentiviral vector was produced by transfection using TransIT-293 transfection reagent (MirusBio) using a third-generation, four-plasmid system of pMDLg/pRRE plus pRSV-Rev, a transfer vector encoding EGFP driven-off of a spleen focus-forming virus promoter, and the plasmid pMD2-G encoding the VSV-G envelope protein. Each flask received a total of 60 μg of plasmid DNA (30 μg of transfer vector, 5 μg of VSV-G, 20 μg of pMDLg/pRRE, and 5 μg of pRSV-Rev) and 180 μl of TransIT-293. Transfection complexes were allowed to form for 15 min at room temperature in 3 ml of Opti-MEM before addition to the flasks. On day 2 posttransfection, media in the flasks were exchanged with 30 ml of fresh complete media. On day 3 posttransfection, the crude viral supernatant was collected at 48 hours posttransfection, and cell debris were removed by centrifugation at 1000*g* for 10 min at room temperature. Supernatants were either used immediately or stored at −80°C.

293-LX cells were plated and transduced at a multiplicity of infection of 0.1. Cells were passaged 72 hours posttransduction and replated at low density. Cells were collected 7 days posttransduction and sorted by GFP fluorescence on an MA-900 (Sony Biotechnology) to positively select the top 30% of EGFP-expressing 293-LX cells. Cells were expanded over two passages, checked for maintenance of EGFP expression, and cryopreserved in CryoStor10.

### Transfection

For lipofection of 293-LX, cells were dissociated and plated at a concentration of 3 × 10^5^ cells/ml (for a final volume of 2 ml for a 6-well plate and 300 ml for a 48-well plate) in complete DMEM medium. Plated cells were allowed to grow overnight in the incubator at 37°C with 5% CO_2_. DNA mixes of purified plasmid DNA (table S8) were prepared in Opti-MEM according to the manufacturer’s protocol. TransIT-293 transfection reagent (MirusBio) was added at ratio of 4 μl reagent to 1 μg DNA, incubated at room temperature for 20 min, and lastly added dropwise across the well. The transfected cells were maintained at 37°C with 5% CO_2_. For all time points exceeding 48 hours, the transfected cells were split 1:3 split at 36 hours to prevent over confluency.

### Nucleofection

Nucleofections of human iPSCs was performed using the Amaxa 4D-Nucleofector unit (Lonza, New Jersey, USA). Nucleofector settings were established empirically (program CB-125, P3 solution) to favor high cell viability and DNA delivery efficiency. Cells were harvested by dissociating cells with Accutase, and once detached, an equal volume of media (plus 10 μM ROCKi) was added and mixed with the cell suspension. Cell numbers were quantified by NucleoCounter (Chemometec, Allerod, Denmark). A total of 800,000 cells per nucleovette condition were centrifuged at 300*g* for 5 min at room temperature. Subsequently, the supernatant was aspirated, and cells were resuspended in P3 solution reagent (10 μl per 800,000 cells). DNA plasmids for nucleofection mixes were prepared in Milli-Q water. Individual nucleovette wells of a 96-well plate condition received a total of 1 μg of DNA mass at a concentration of 250 ng/μl and 6 μl of P3. A 10-μl aliquot of the P3 cell suspension was added to each nucleovette before nucleofection. After executing the nucleofection program, cells were incubated at room temperature for 10 min and then resuspended with 80 μl of media (with 10 μM ROCKi) for a total volume of 100 μl per nucleovette. Cells were seeded at 120,000 cells for 96-well and 170,000 cells for 48-well formats, respectively. To decrease apoptosis in transfected iPSCs, 100 μM Z-VAD FMK was added to the media for the first 24 hours, and a BCL-XL expression plasmid was included in the DNA mix (*34*). Posttransfection, cells were incubated for 24 hours at 37°C with 5% CO_2_ in a humidified incubator.

### Flow cytometry

Single-cell suspensions were prepared by detaching the cells with TrypLE and resuspended in DMEM media. Subsequently, cells were washed in the following cell staining buffer (CSB): 2% FBS, 0.5 mM EDTA, and 0.4% NaN_3_. For CXCR4 and CD105 detection, cells were stained with Brilliant Violet 421 anti-human CD184 (CXCR4) and allophycocyanin (APC) anti-human CD105 at a 1:100 concentration. For DLL4, CD4, CD105 detection, cells were stained with phycoerythrin (PE)/Dazzle 594 anti-human CD4 (1:600), PE anti-human DLL4 (1:200), and Brilliant Violet 421 anti-human CD105 (1:600), all from BioLegend (San Diego, CA, USA). Antibody incubation was for 30 min at 4°C in the dark. Flow cytometry antibody staining for iPSCs included additional steps: After iPSCs were dissociated and washed once, they were incubated for 20 min on ice with an Fc-blocking step using immunoglobulin G (IgG) human serum diluted 1:400 in CSB. The iPSCs were centrifuged at 500*g* for 5 min, followed by decantation, and addition of antibody solution. For the live-dead stain, cells were resuspended in CSB containing DRAQ7 (1:1000) or with propidium iodide at 1 μg/ml. Cells were analyzed with either a Cytoflex (Beckman Coulter) or NovoCyte Penteon (Agilent) flow cytometer. Data analysis was performed using FlowJo v10.10.0. At least 3 × 10^5^ singlet, live cells were counted for each sample.

### Generation of virtual library of nongenome targeting CTS

See fig. S12 for the schematic illustration of the overall process. Ensembl versions of genomes at release 103 were acquired for human (homo_sapiens), mouse (mus_musculus), cow (bos_taurus), atlantic salmon (salmo_salar), chicken (gallus_gallus), and pig (sus_scrofa). The Pacific bluefin tuna genome was acquired from the National Center for Biotechnology Information (NCBI) with GenBank accession number GCA_009176245.1 (*Thunnus orientalis*, tuna2). Blast databases were created for each genome using ncbi-blast version 2.12. A fasta of 2 million random CTS sequences was created using RandomSeq (*30*) with settings FLS, *n* = 2,000,000, cap_start = False, cap_stop = True, fasta = True, allow_start = False, allow_stop = True, stop_codons = “AGG,TGG,CGG,GGG,” length = 20. This generates a compendium of 23-bp CTS with the four potential NGG Cas9 recognition sites at their 3′ ends. NCBI blastn was run against these genomes sequentially with the 2 million CTS using default settings and task “blastn-short” which is optimized for sequences shorter than 50 bases. Using data from the foundational GuideSeq paper (*64*) as a reference point bona fide off-target sites harbored as many as six mismatches within the protospacer sequence. Hence, when CTS sequences were aligned to each genome, they were only included in the next alignment if they had seven or greater mismatches and additionally less than a 16-base stretch of match. In addition, a set of heuristics based on a literature study of off-target analyses was used to filter these sequences further. Sequences with higher G-content in the nonseed region distal from the PAM motif have been shown to have stronger off-target activity (*31*). Hence, all sequences with greater than or equal to five Gs in the 10-bp most distal to the PAM were discarded. Thymidines in the 3′ region of spacer are disfavored for both Cas9 loading and cleavage efficiency (*33*); hence, all sequences with three or more Ts in the region directly adjacent to the PAM were discarded. Since extreme degrees of GC content tend to hinder guide RNA function (*33*), only CTSs with GC content between 35 and 75% were retained (*32*). In addition, since our backbone plasmid contains one Eco RI, two Bsm BI, and one Sap I restriction enzyme sites that are used for downstream cloning, any CTSs that generated additional instances of these sites within the backbone were discarded. Following alignment and filtering of CTSs, we were left with a list of >50,000 CTS sequences.

### RNA-seq of guide RNAs

GFP–293-LX cells in six-well plates were transfected with Cas9 and a proGuide, with or without the trigger guide. Thirty-six hours posttransfection, cells were detached and counted. One million cells were directly lysed in 300 μl of Tri-Reagent (Zymo Research) and stored at −80°C. Total RNA was extracted using the Direct-zol RNA Miniprep Plus kit (Zymo Research) according to the manufacturer’s guidelines. cDNA of the guide RNAs was synthesized with the Template Switching RT Enzyme Mix (New England Biolabs) using the 2nd Strand cDNA Synthesis Protocol. Specificity of guide RNA synthesis was ensured by using a 3′ primer specific for the 3′ end of the guide RNA scaffold present in all fully transcribed guide RNAs. Briefly, 800 ng of total RNA was used for annealing of the 3′ primer in a total volume of 24 μl and incubated for 5 min at 70°C. For reverse transcription, 9 μl of the annealing mixture was added to 6 μl of the RT mix and incubated at 42°C for 90 min, followed by 5 min at 85°C. For the subsequent synthesis of the second strand, 10 μl of the RT mix was added to 90 μl of the second-strand cDNA synthesis reaction mix and incubated at 37°C for 15 min, then at 95°C for 1 min, and 65°C for 10 min. Last, 30 μl of this mixture was cycled for 20 polymerase chain reaction (PCR) cycles to amplify the cDNA for the guide RNA and sequenced using an Illumina MiniSeq, generating 150-bp paired-end reads.

Fastqs of the RNA-seq data following PCR of guide sequences were assembled and R1 and R2 filtered to remove triggerGuide sequences (which share the same scaffold sequence). Reads were also filtered to remove both for very short (<50 bp) and very long reads (>160 bp). Reads were modified with cutadapt to remove CS1 (ACACTGACGACATGGTTCCTACAGGG) and CS2 (AGACCAAGTCTCTCGTACCGTA) linkers, allowing one mismatch. Flash was used to combine R1 and R2 with min-overlap 10. Crispresso2 was run on all architectures (see table S2 for settings). Perfect adjacent repair is a heuristic of repair outcomes that are “close” to perfect repair but accepting of sequencing error or slight differences in size. A perfect adjacent repair is a nonperfect repair that has at most five substitutions, one and only one deletion within 3 bp of a perfect repair site, and up to four additional deletions of which all must be under 5 bp, of which only two can be above 2 bp. Inversions were detected with crispresso2 using the architecture if the excised DNA was placed in reverse in a perfect repair.
