# Supplementary material for: Programmable multistep CRISPR gene activation via control of RNA polymerase III termination
Source: Sci Adv. 2025 Dec 5;11(49):eadt1532. doi: 10.1126/sciadv.adt1532 (PMC12680031; doi:10.1126/sciadv.adt1532)
Supplement: Supplementary file 1 — Figs. S1 to S17 Legends for tables S1 to S8 [file sciadv.adt1532_sm.pdf]

Science Advances
AAAS

Supplementary Materials for

Programmable multistep CRISPR gene activation via control of RNA polymerase III termination

Anupama K. Puppala *et al.*

Corresponding author: Bradley J. Merrill, merrillb@uic.edu; Ryan Clarke, ryan@cellgorithm.com

Sci. Adv. **11**, eadt1532 (2025)
DOI: 10.1126/sciadv.adt1532

The PDF file includes:

Figs. S1 to S17
Legends for tables S1 to S8

Other Supplementary Material for this manuscript includes the following:

Tables S1 to S8

Supplementary Text

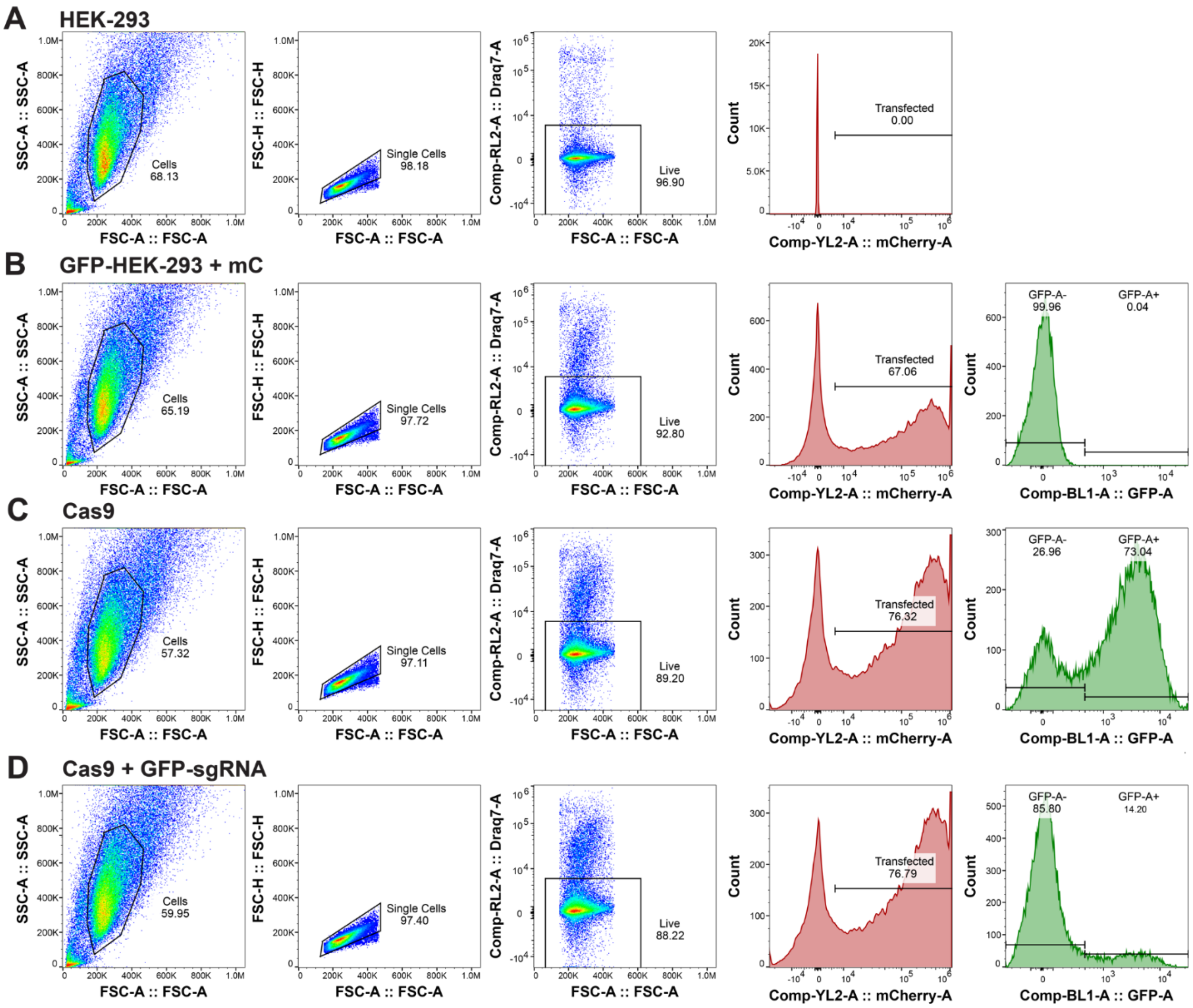

Fig. S1. Gating strategy for GFP disruption experiments. HEK293T cells were first identified by SSC-A vs. FSC-A gating, and single cells were subsequently isolated based on FSC-A vs. FSC-H. Because HEK293T cells generally exhibited high viability, the live-cell gate was determined using heat-killed HEK293T samples to identify cells with low DRAQ staining. After establishing these gates, transfected cells were selected by gating on mCherry-positive cells. Representative plots showing **(A)** Untransfected, non-GFP, HEK293 cells. **(B)** GFP-transgene containing HEK293 cells transfected with mCherry expression plasmid. (**C**) GFP-transgene containing HEK293 cells transfected with a Cas9 plasmid without an sgRNA to define the GFP-A$^-$ and GFP-A$^+$ populations. **(D)** Addition of an sgRNA plasmid targeting GFP to condition in (C).

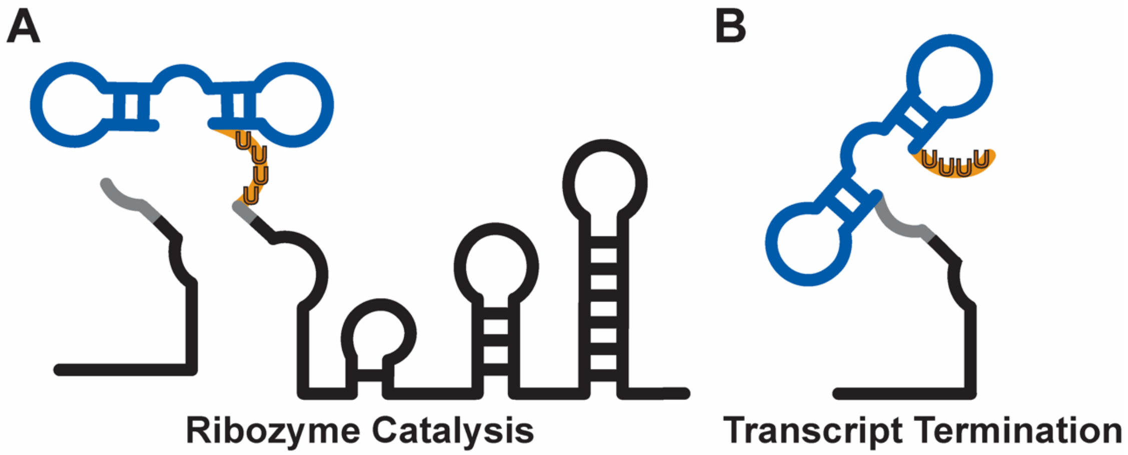

Fig. S2. Schematic of previous combined methods for inactivating RNA to make proGuides. Illustrations of the RNA transcripts resulting from the redundant inactivation elements incorporated into a tetraloop region (gray). Inactivation elements encoded a hammerhead ribozyme (blue) and an RNA Pol III termination signal of six contiguous thymidine residues (orange). (**A**) Ribozyme-based inactivation method utilizes cleavage of the hammerhead ribozyme such that the upstream and downstream sequences dissociate into two separate RNA strands. (**B**) The transcript termination method prevents transcription of necessary functional elements of the guide RNA (i.e. hairpin 1, hairpin 2) by placing a termination signal upstream of those elements in the proGuide.

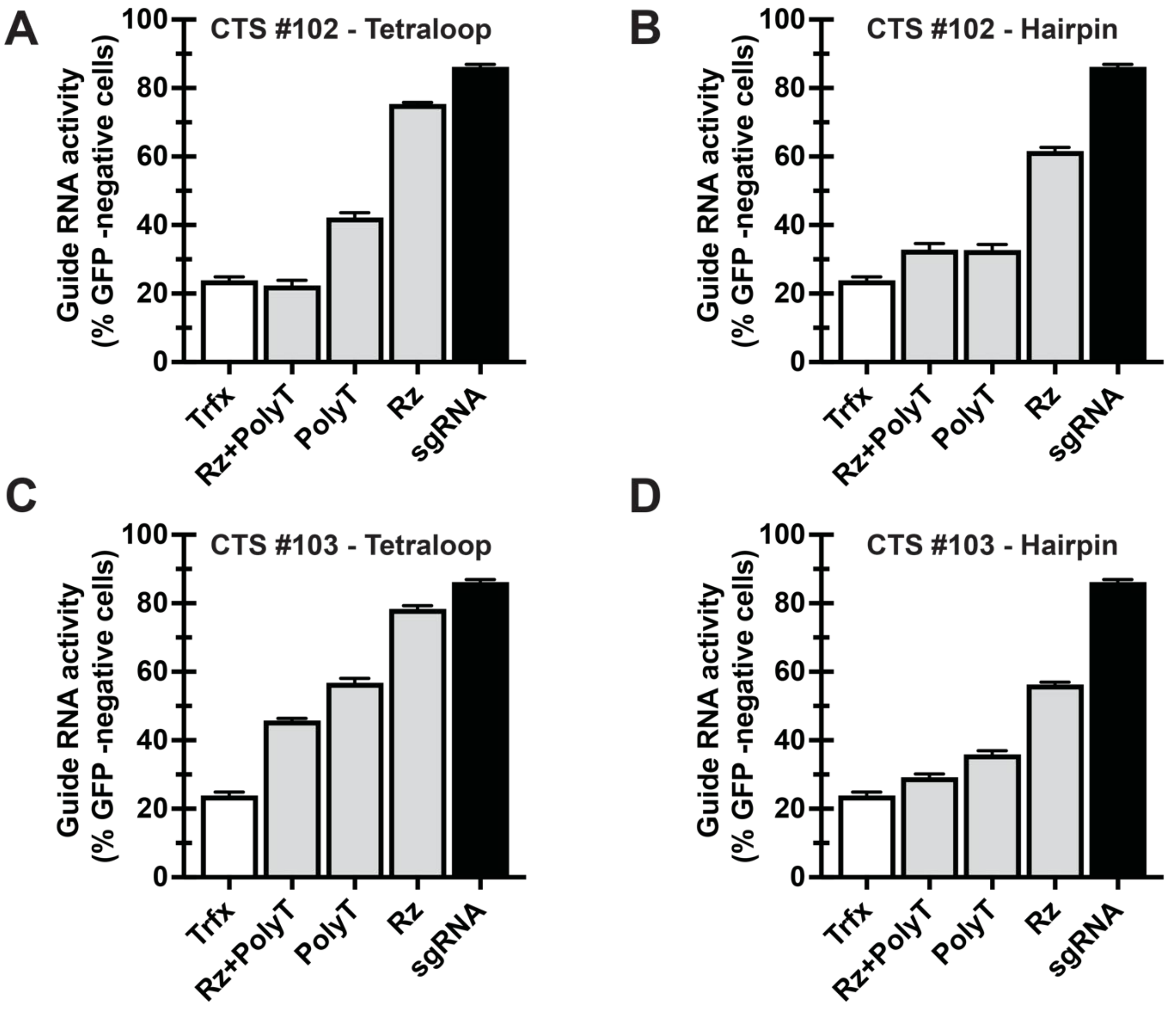

Fig. S3. Poly T tract alone is more effective than ribozyme alone at inactivating proGuides. Data are similar to those described in Fig. 2C with the exception that different CTS flank the inactivation moiety. (**A, C**) CTS102 and CTS103 flank the inactivation moiety within the gRNA tetraloop, respectively. (**B, D**) CTS102 and CTS103 flank the inactivation moiety within hairpin 1 of the gRNA, respectively. Residual activity of proGuides in cells was primarily affected by the inactivation unit (ribozyme vs. polyT tract) and insertion location (hairpin vs. tetraloop) and minimally affected by the CTS sequence.

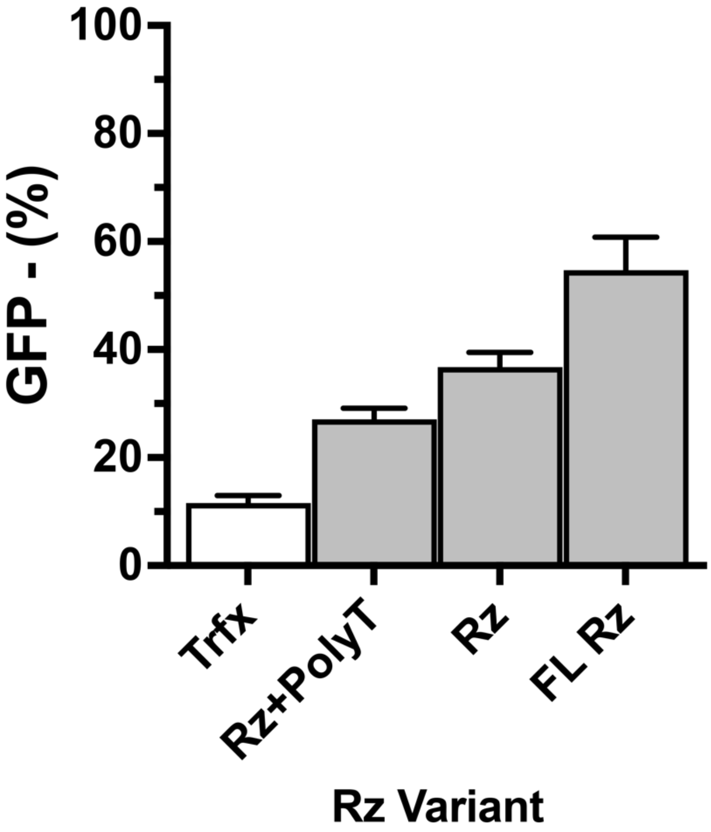

Fig. S4. A full-length hammerhead ribozyme is ineffective as an inactivating unit for proGuides. Similar to experiments shown in Fig 2C. For a given proGuide, different inactivation units generate different levels of leak of GFP disruption in the absence of a trigger guide. Ribozyme only inactivation units led to a larger level of leak. The full-length hammerhead Rz, which exhibits higher *in vivo* nucleolytic activity, displayed the highest level of unwanted proGuide activity.

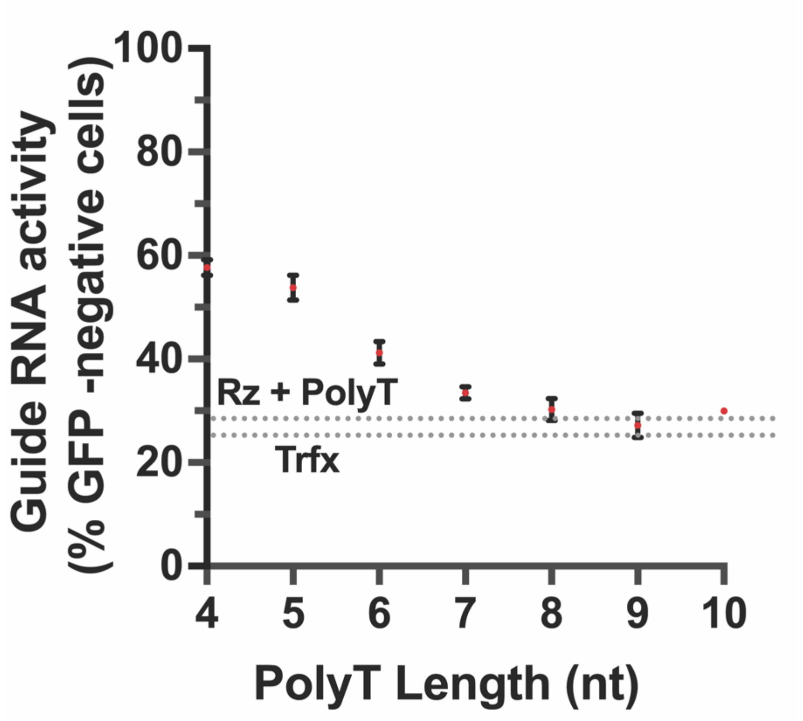

Fig. S5. Increasing length of the polyT tracts embedded in hairpin 1 reduces proGuide leakiness. Similar to the experiment shown in Fig 2D, except polyT tracts were inserted into hairpin 1 instead of into the tetraloop.

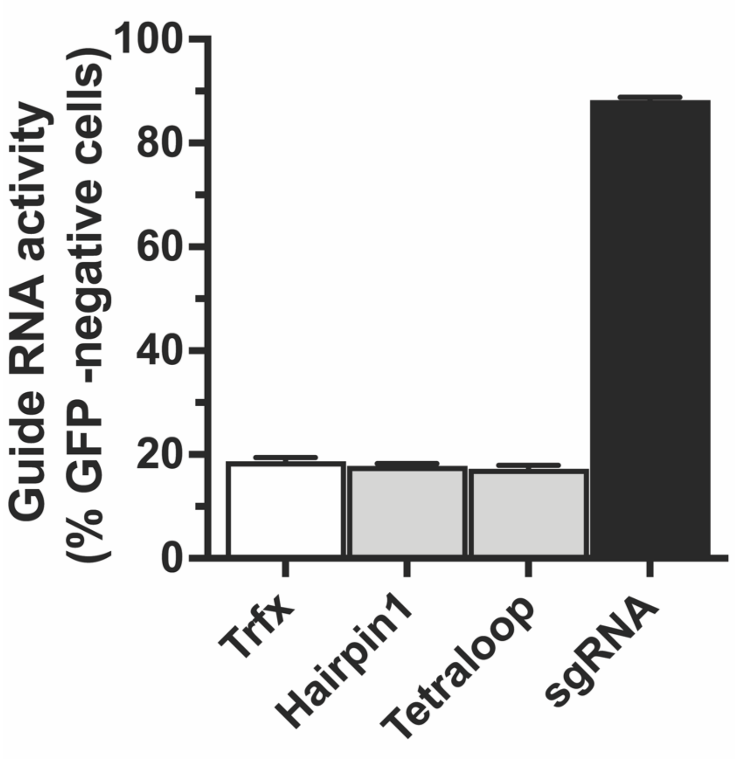

Fig. S6. Early terminated guide RNAs are inactive. RNA transcripts comprising only the 5' portion of the proGuide sequence upstream of the polyT termination tract at either the tetraloop site or the hairpin 1 site (gray bars) exhibit no residual Cas9 cleavage-competency in cells compared to transfected cells (white bar).

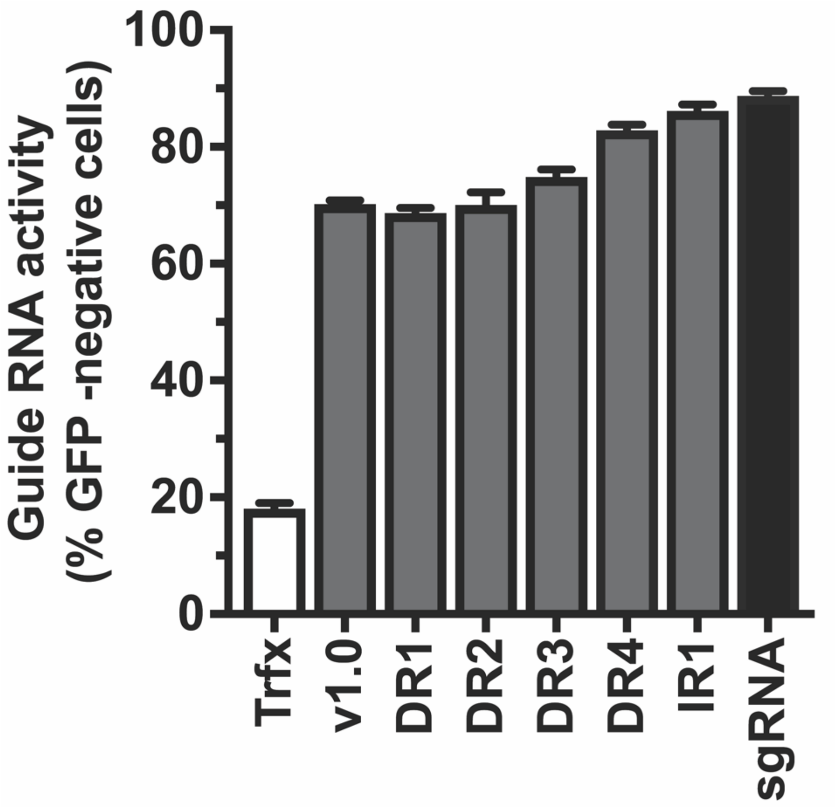

Fig. S7. The inverted repeat CTS configuration confers efficacy similar to an sgRNA. Similar to the experiment shown in Fig. 3B, except inactivation sequences were inserted into the hairpin 1 site instead of the tetraloop of proGuide constructs.

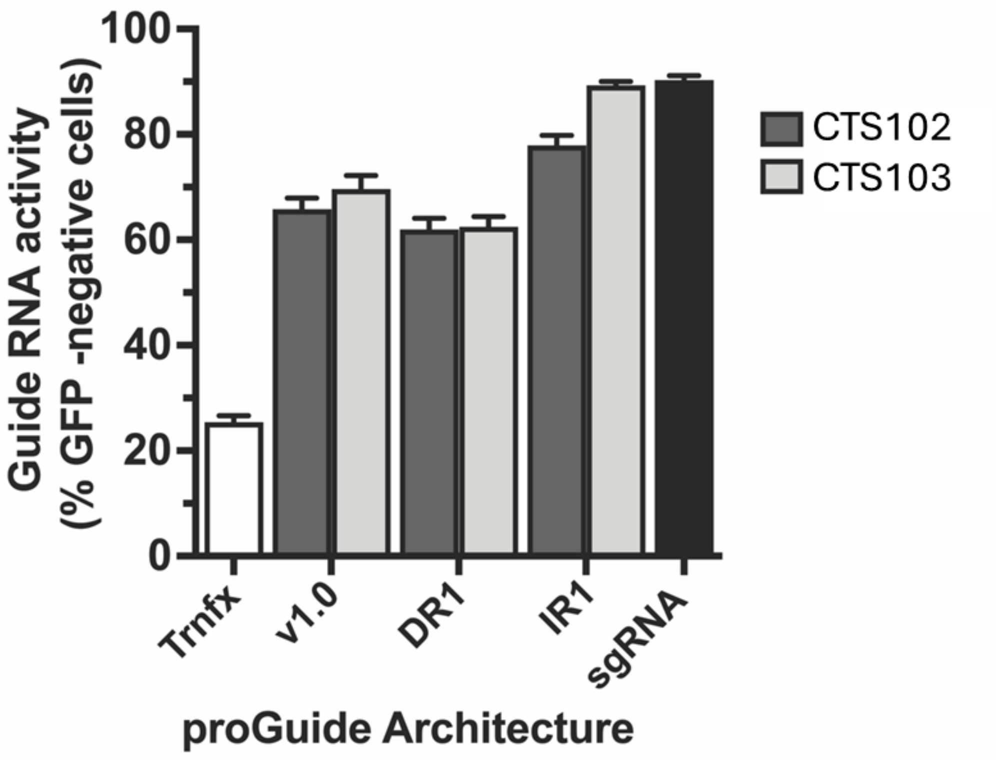

Fig. S8. Inverted repeat configuration outperforms direct repeat configuration for multiple CTS sequences. Similar to the experiment shown in Fig. 3B, except proGuides had CTS102 and CTS103 sequences instead of CTS101.

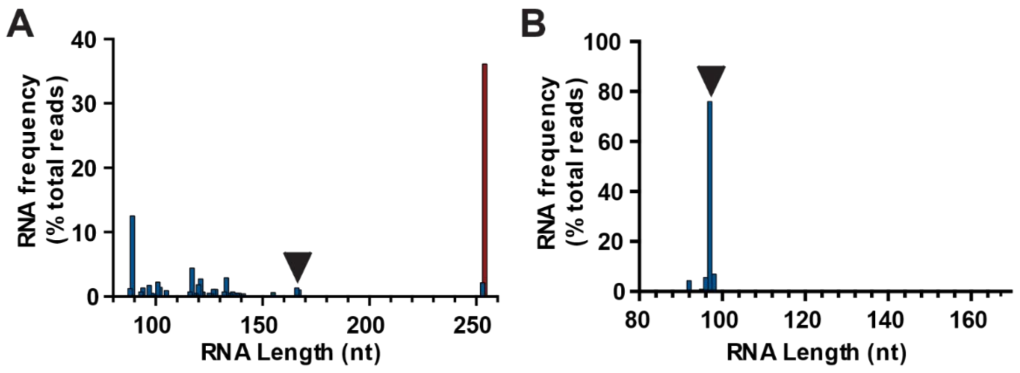

Fig. S9. Analysis of RNA produced from proGuide plasmids following co-transfection with trigger sgRNA. (**A**) RNA was isolated from HEK293T cells transfected with a trigger sgRNA, Cas9 plasmids and the previously published proGuide architecture containing a ribozyme and CTS102 sites. Oligos specific to the proGuide spacer sequence and the 3' end of the common guide RNA sequence were used to amplify cDNA made from isolated RNA. DNA sequencing of amplicon products were analyzed with Crispresso2, and the graph shows the distribution of fragment lengths that mapped to the proGuide sequence. The arrowhead indicates the size of an expected perfect repair outcome, where the ends of the two CTS are repaired via NHEJ. (**B**) Same procedure as in (A), except the proGuide harbored the polyT tract inactivation element and higher efficiency CTS101 sites in an inverted repeat orientation.

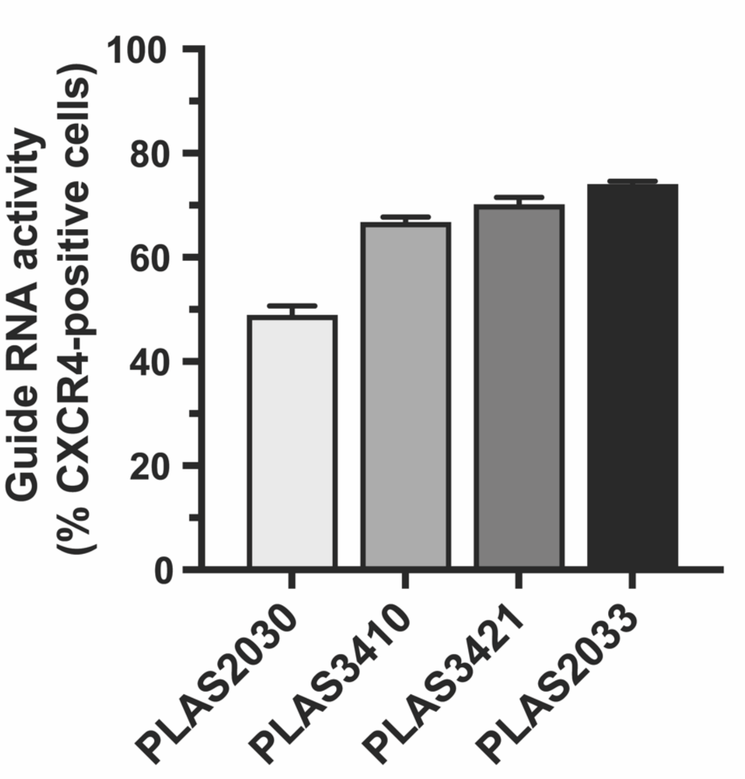

Fig. S10. Evaluation of CXCR4-targeting 14 nt spacer sequences for activation of CXCR4. Frequency of cells expressing CXCR4 protein on the cell surface was measured by flow cytometry 48 hr after transfection with plasmids encoding Cas9-VPR and a CXCR4-targeting sgRNA. Each guide RNA plasmid used a different 14 nt sequence for the spacer targeting CXCR4 promoter region. PLAS2033(GGAAGGAGGGCGGCA), PLAS3410(CTGCTGTTTGCGGG), PLAS3421(AACGCGTCTCTCTG), PLAS2030(GCGGGGAATGGCGT)

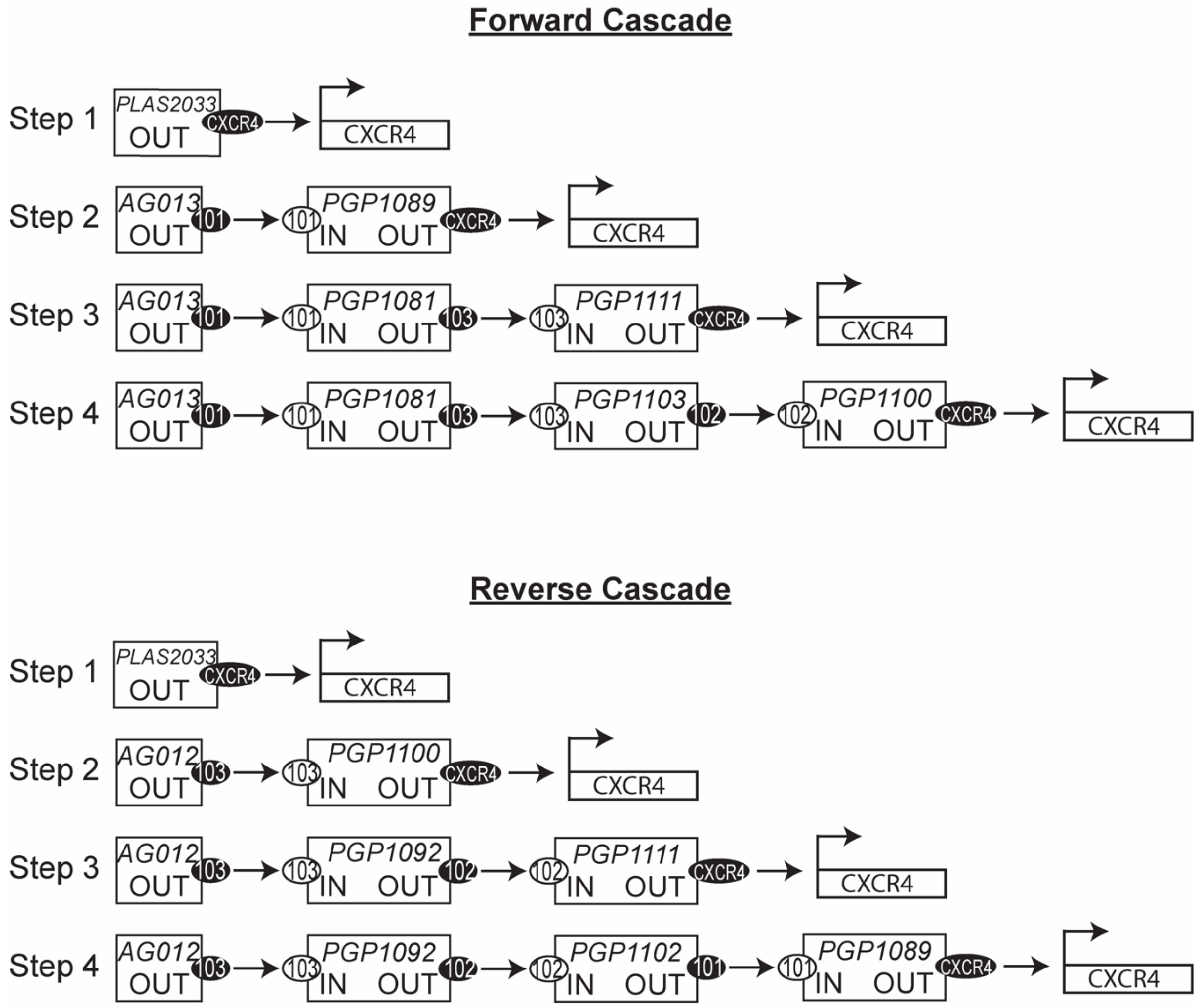

Fig. S11. Schematic of proGuide cascades resulting in the transcriptional activation of an endogenous gene. Following the design of the cascade schematic illustrated in Fig. 2A, each row depicts the arrangements of plasmid DNA delivered via transient transfection to HEK293 cells for graphs shown in Fig 5C. PLASxxxx and PGPxxxx rubric corresponds to plasmid DNA unique identification labels for guide RNA and proGuide expression plasmids, respectively. The numbers within ovals (101, 102, 103) refer to the CTS sequences (white) and spacer sequences (black) present in each plasmid DNA. The black oval containing CXCR4 indicates the presence of a 14nt spacer sequence targeting the CXCR4 promoter region for CRISPRa transcriptional activation by Cas9-VPR.

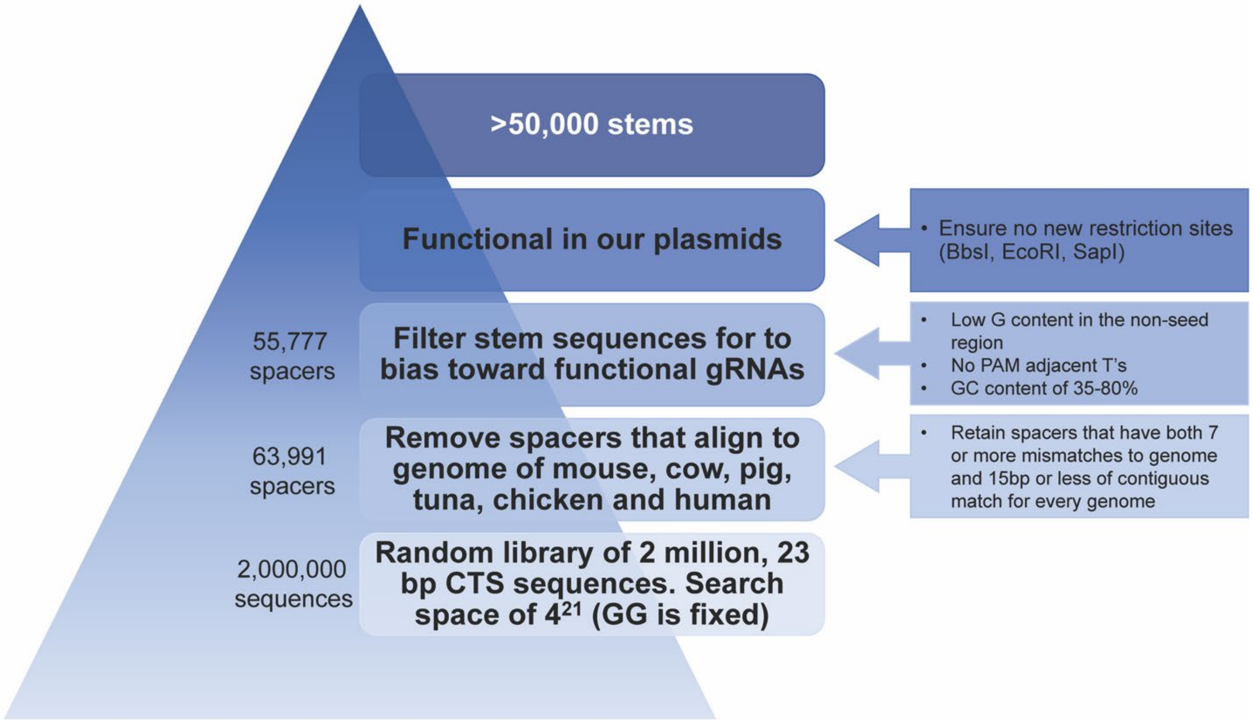

Fig. S12. Schematic for the process of generating virtual library of CTS sequences for proGuides. See Materials and Methods for description.

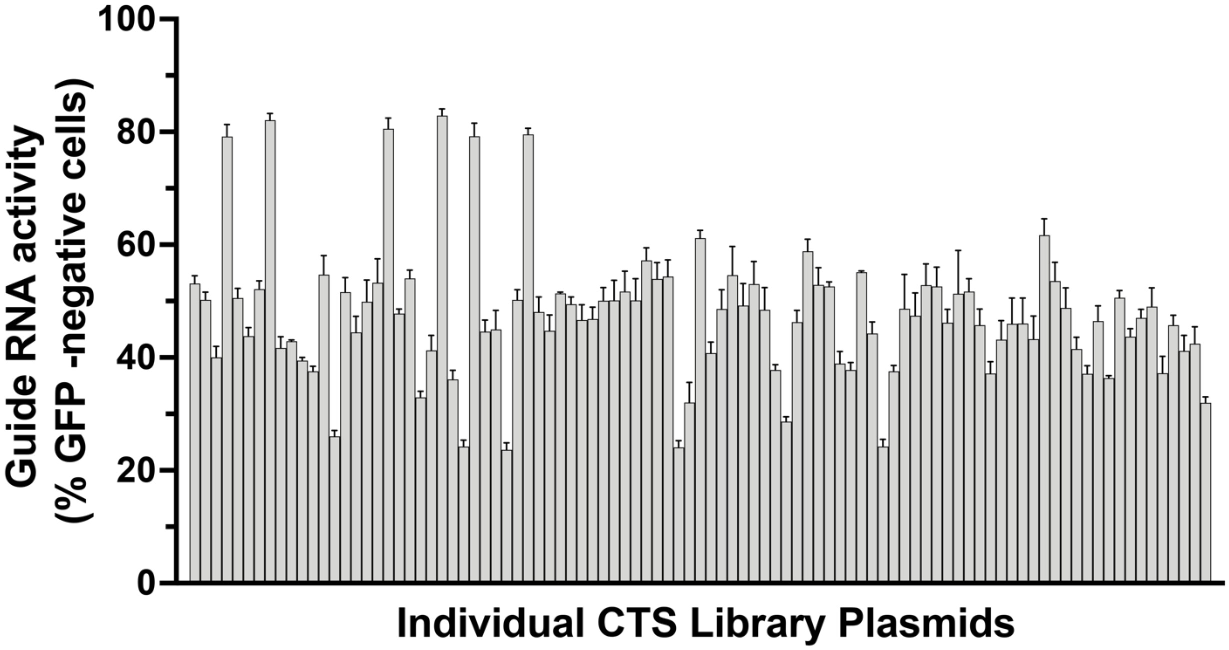

Fig. S13. Screen of 96 CTS sequences for conversion to an active guide RNA. Individual plasmid DNA expressing both an EGFP-targeting proGuide and a trigger guide RNA targeting the CTS sequences in the corresponding proGuide were constructed. A plasmid library of approximately 10,000 sequences from Fig. S12 was generated, from which 96 individual plasmid DNA were isolated and evaluated. Disruption of EGFP was measured two days after transient transfection. Note that DNA sequencing of plasmids showed that the six very high activity plasmids were defective proGuide cloning artifacts, and the five very low activity plasmids harbored defective trigger RNAs. Excluding these sequences, the CTS sequences exhibiting the highest GFP knockdown were subsequently used to construct proGuides evaluated in Fig. 5D.

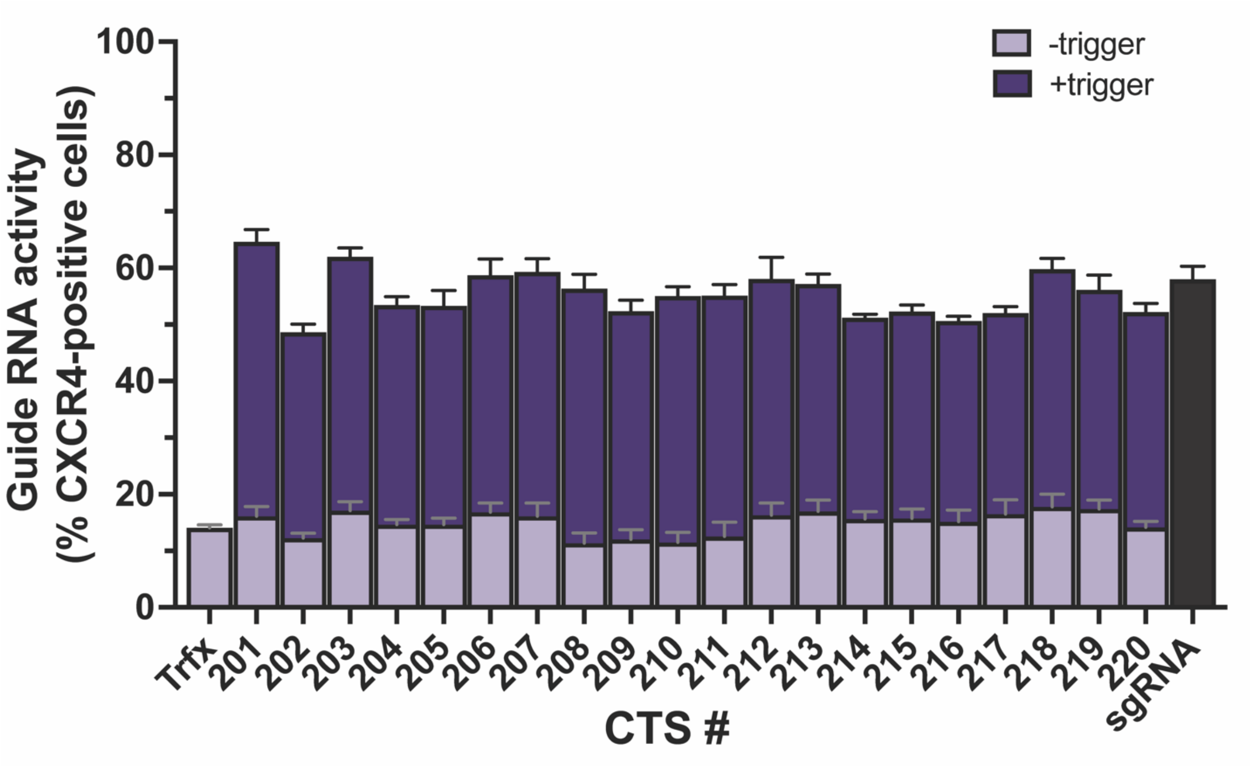

Fig. S14. Evaluation of top CTS for proGuides using CRISPRa activation of CXCR4 surface protein expression. Related to Fig. 5D, except proGuides contained 14 nt spacer sequences targeting the CXCR4 promoter. Transient transfection of the proGuide plasmids and a Cas9-VPR expression plasmid enabled increased CXCR4 expression only with expression of the trigger gRNA.

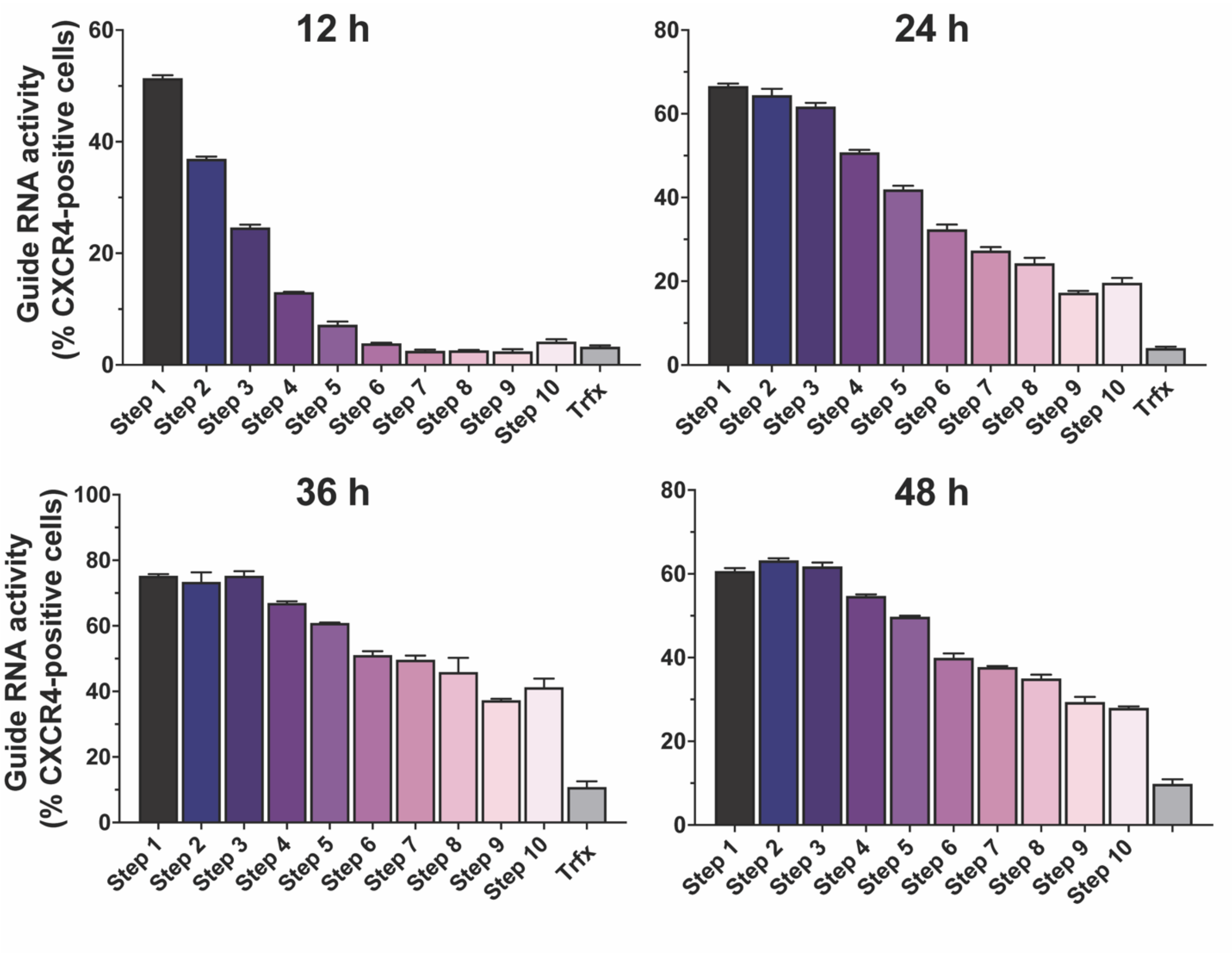

Fig. S15. Evaluation of kinetics of a 10-step cascade of proGuides. The forward core cascade from Fig 5E was transfected into HEK293T cells with a Cas9-VPR and a proGuide for activation of CXCR4 at the indicated step. Flow cytometry for surface CXCR4 protein expression was performed at the indicated times after transfection.

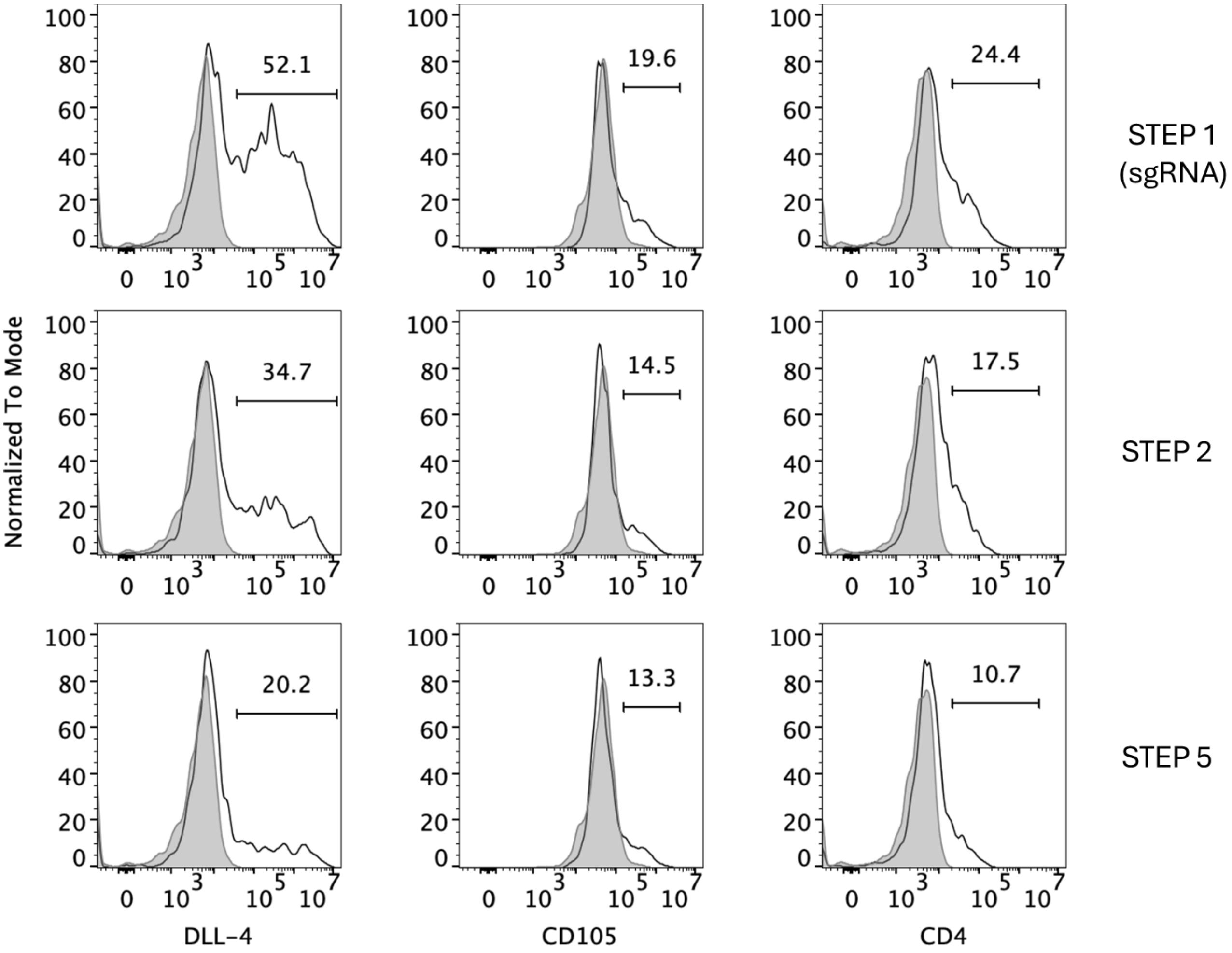

Fig. S16. Intensity and frequency of cell surface marker expression in iPSC by CRISPRa with sgRNA or proGuides. Frequencies of the indicated cell surface marker (DLL4, CD105, CD4) intensities is displayed as percent maximal count scaled to all channels. The white line represents iPSC nucleofected with guide for activation of the marker (listed at bottom) programmed at different steps (listed on right), and it overlays control cell (gray shadow) nucleofected with core cascade without CRISPRa guide RNA. Plots show all live transfected cells 48hrs after nucleofection with plasmid DNA cascades illustrated in Fig. 8. Numbers indicate percentage of cells within the gate (brackets) identified as positive for surface marker expression. Plots are individual samples representative of triplicates transfections.

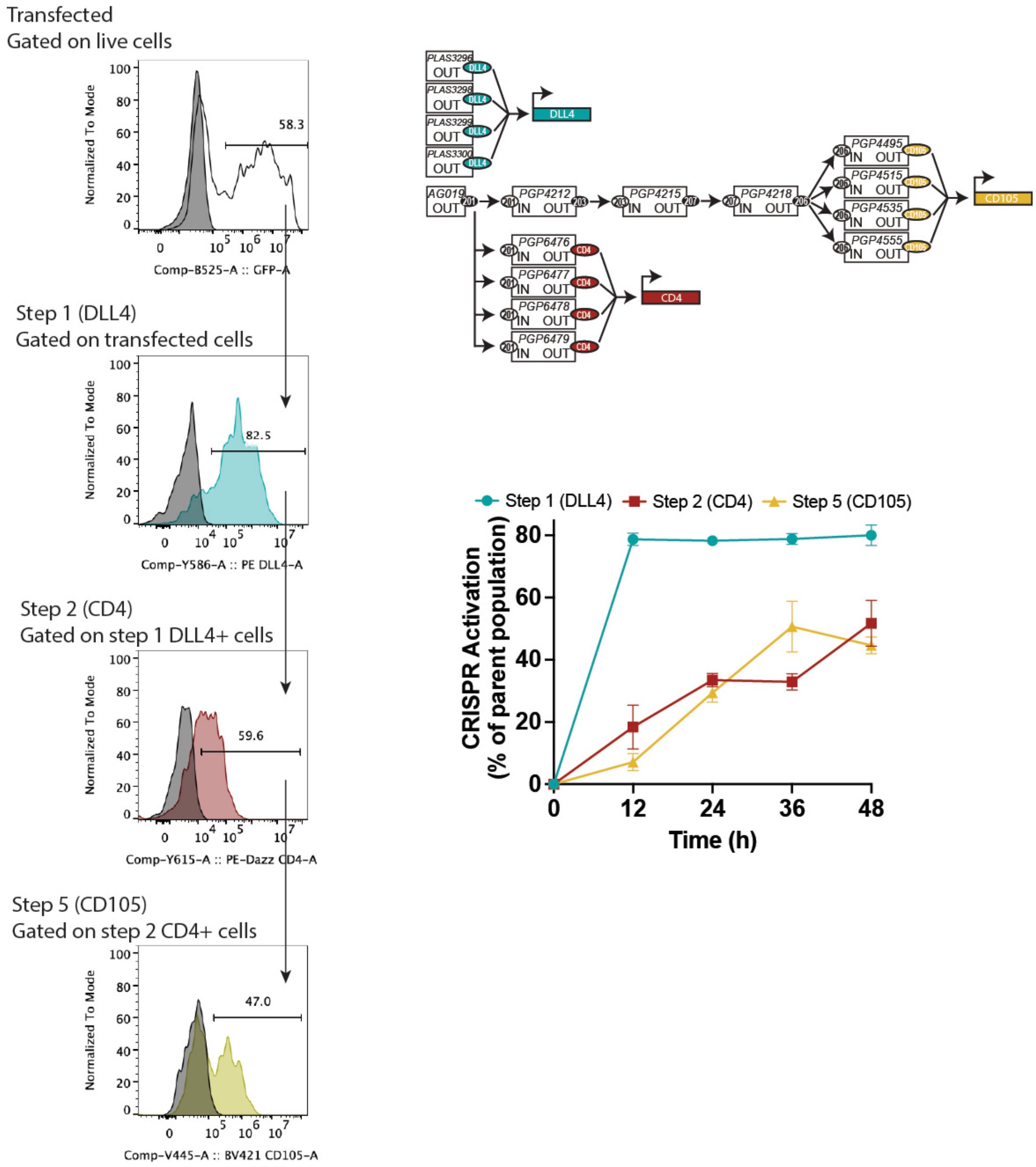

Fig. S17. Gating strategy for assessing progression through sequential steps of a proGuide cascade. (Left) The series of gating steps starting with live, transfect cells is used for the input of the next gate based on the programmed order of surface marker gene activation from the cascade of proGuides (**Fig 8**). Numbers represent the percentage of the parental population that is positive for the indicated marker. The examples shown are for the 48hr time point for cells nucleofected with the cascade of proGuides shown on the right. The gating strategy was used to generate graphs for **Fig 9**.

Table S1. Complete DNA sequences for plasmids use in this study.

Table S2. NGS repair outcome data, summary of analyses, and tables for classification of repair for all CTS orientations in Fig 4.

Table S3. NGS repair outcomes for DR1 orientation in Fig 4.

Table S4. NGS repair outcomes for DR2 orientation in Fig 4.

Table S5. NGS repair outcomes for DR3 orientation in Fig 4.

Table S6. NGS repair outcomes for DR4 orientation in Fig 4.

Table S7. NGS repair outcomes for IR1 orientation in Fig 4.

Table S8. DNA mass compositions used for transfection of proGuide plasmid mixtures
